# Functional excitation-inhibition ratio indicates near-critical oscillations across frequencies

**DOI:** 10.1162/imag_a_00318

**Published:** 2024-10-17

**Authors:** Marina Diachenko, Additya Sharma, Dirk J. A. Smit, Huibert D. Mansvelder, Hilgo Bruining, Eco de Geus, Arthur-Ervin Avramiea, Klaus Linkenkaer-Hansen

**Affiliations:** Department of Integrative Neurophysiology, Center for Neurogenomics and Cognitive Research (CNCR), Amsterdam Neuroscience, VU Amsterdam, Amsterdam, The Netherlands; Department of Psychiatry, Amsterdam University Medical Center, University of Amsterdam, Amsterdam, The Netherlands; Amsterdam Neuroscience, Amsterdam, The Netherlands; Amsterdam Brain and Cognition, University of Amsterdam, Amsterdam, The Netherlands; Department of Child and Adolescent Psychiatry, Amsterdam UMC, University of Amsterdam, Amsterdam, The Netherlands; Department of Biological Psychology, Vrije Universiteit Amsterdam, Amsterdam, The Netherlands

**Keywords:** critical brain dynamics, detrended fluctuation analysis, excitation-inhibition balance, neuronal oscillations

## Abstract

The concept of excitation/inhibition (E/I) balance plays an important role in understanding brain function in health and disease. We recently introduced an algorithm to determine a functional E/I ratio based on the critical brain dynamics that emerge in neuronal networks balancing between order and disorder. Little, however, is known about the frequency specificity of E/I regulation and how to measure it. Here, we optimized the algorithm for measuring functional excitation-inhibition ratio (*fE/I*) in narrow frequency ranges and validated it on a computational model of critical oscillations and EEG data. In the computational model, we confirmed that*fE/I*discriminated E/I connectivity differences across a wide range of frequencies (1–150 Hz). Twin EEG data revealed significant genetic influences on*fE/I*across frequencies, whereas contrasting eyes-open and -closed EEG indicated functional changes of*fE/I*restricted to a subset of alpha and beta oscillations and brain regions. We propose that assessing*fE/I*with finer frequency resolution will prove useful for understanding the functional role of E/I regulation in a spectrally refined fashion in health and disease.

## 1 Introduction

Balance between neuronal excitation and inhibition (E/I balance) is the key mechanism for maintaining critical brain dynamics on multiple levels of neuronal organization, such as scale-free variability in the size of neuronal avalanches (Beggs & Plenz, 2003) or power-law scaling of long-range temporal correlations of neuronal network oscillations (Buzsáki, 2006;Linkenkaer-Hansen et al., 2001,^2007^;Palva et al., 2013;Smit et al., 2011). Computational studies indicate that these critical phenomena co-emerge at the same E/I balance (Poil et al., 2012). Importantly, criticality of both neuronal avalanches (Gautam et al., 2015;Kinouchi & Copelli, 2006;Shew et al., 2009) and oscillations (Avramiea et al., 2020) has been associated with optimal processing of stimuli (Beggs, 2008). Critical networks with balanced E/I exhibit maximal information transfer capacity achieved through phase and/or amplitude coupling (Avramiea et al., 2022). These findings suggest that alterations in the intricate interplay between excitatory and inhibitory forces in neuronal circuits may interrupt critical dynamics of neuronal networks and impair their function. Indeed, analyses of oscillations have implicated E/I imbalances with various brain disorders, including Alzheimer’s disease (van Nifterick et al., 2023), STXBP1 syndrome (Houtman et al., 2021), autism spectrum disorder (Bruining et al., 2020;Uzunova et al., 2016), epilepsy (van van Hugte et al., 2023), and schizophrenia (Lisman, 2012;Liu et al., 2021). Interestingly, E/I imbalances in these disorders associate with various mechanisms and a certain degree of spectral specificity of the pathophysiology. This calls for quantitative measures of E/I balance to capture functionally relevant changes in brain oscillations across different frequency ranges.

We recently developed an algorithm to quantify E/I balance at the network level from ongoing oscillations termed functional E/I (*fE/I*) ratio (Bruining et al., 2020). The*fE/I*measure correlates strongly with the level of criticality of a neuronal network in computational models and is capable of detecting E/I shifts associated with pharmacological manipulation in human EEG. Typically, the algorithm has been applied to particular traditionally defined frequency bands such as alpha (Geertjens et al., 2022;Juarez-Martinez et al., 2022,^2023^;Li et al., 2022;van Nifterick et al., 2023). However, some studies adopt narrower frequency bins (Fuscà et al., 2023;Houtman et al., 2021;Javed et al., 2023;Kat et al., 2024;Stuiver et al., 2024;S. H. Wang et al., 2024). This latter approach can uncover distinct spectral patterns that may lie outside the canonical frequency-band paradigm, particularly when investigating pathologies, conditions, or species with limited or no a priori knowledge about the type of neuronal oscillations that are affected. It should, however, be noted that various parameters of the*fE/I*algorithm, such as the filter settings and fitting range, have not been calibrated and validated for application in narrow frequency bins.

Here, we optimize the*fE/I*algorithm for use in narrow ranges across the full frequency span. We tune the spectral and temporal parameters of the algorithm for concurrent application across the spectrum by considering the spectral and temporal characteristics of underlying filters. Using data from the critical oscillations model, we show that*fE/I*can detect modulations in E/I connectivity across a wide range of frequencies. We support the biological significance of this spectral*fE/I*by estimating to what extent it is affected by genes, using EEG data from twins. Finally, we test the capability of*fE/I*to detect spectrally confined E/I changes between eyes-open and eyes-closed brain states. To support the wide use of our techniques, we offer a free-access Python/C implementation of the algorithm. Overall, the fine-grained application of*fE/I*across the frequency spectrum is likely to provide novel insights into how disease affects specific neuronal circuits.

## 2 Methods

### 2.1 Detrended fluctuation analysis

**Fig. 1.**
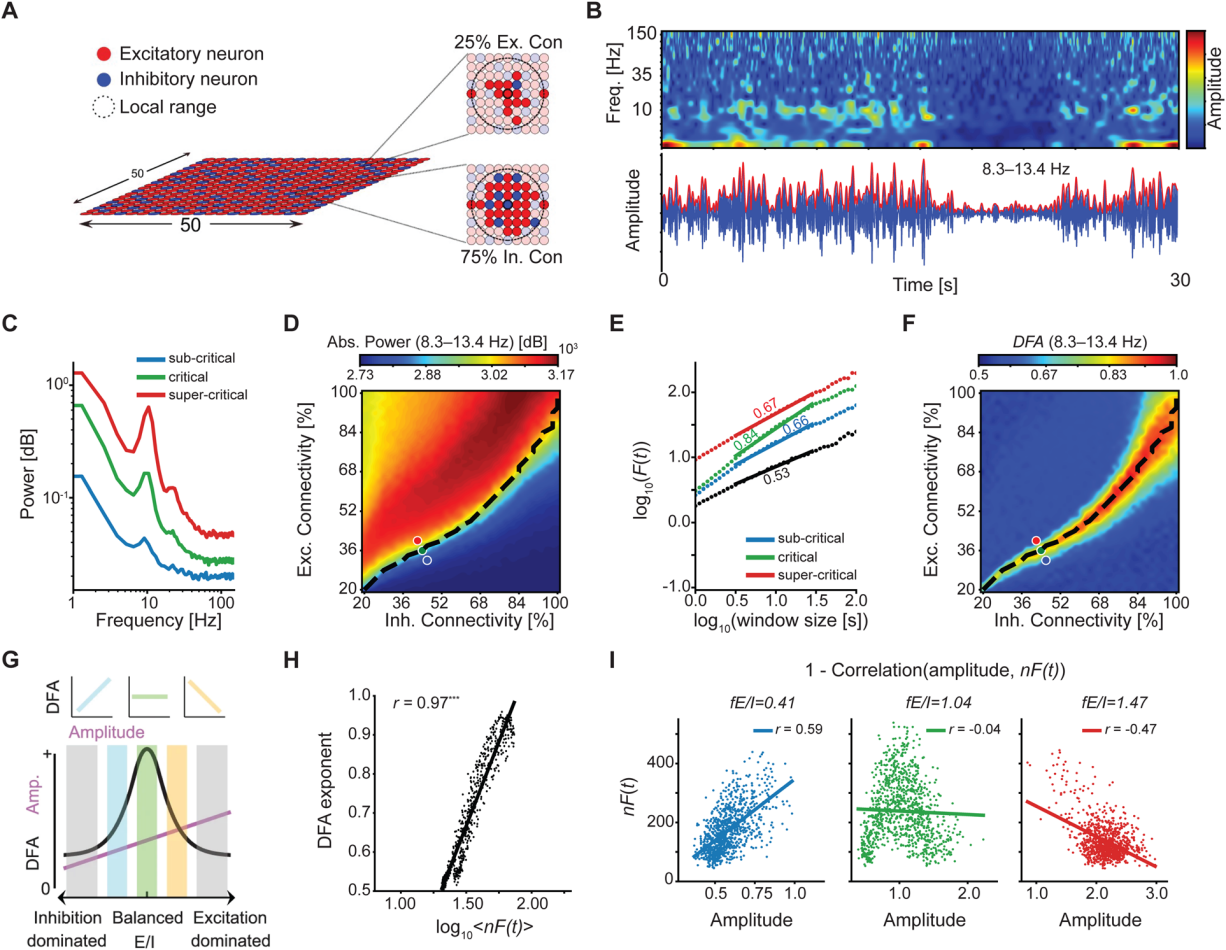
Rationale of the network-level measure of functional E/I ratio. (A) The CROS model, consisting of 75% excitatory and 25% inhibitory integrate-and-fire neurons arranged on a 50 x 50 open grid, produces (B) oscillatory activity similar to EEG. Changing the ratio of percentage of excitatory and inhibitory synapses that each neuron connects to within its local range of the CROS model affects (C, D) the amplitude and (E, F) temporal structure of oscillations produced. An increase in excitatory connectivity relative to inhibitory connectivity leads to an increase in signal power as shown for the three networks marked by*blue*(sub-critical network),*green*(critical network), and*red*(super-critical network) circles in (D). The*dashed black*line in (D) and (F) corresponds to critical neuronal avalanches (*k*= 1), which overlaps with networks with the strongest long-range temporal correlations. We define balanced connectivity ratios as those supporting the critical dynamics of avalanches and oscillations. Oscillations can exhibit long-range temporal correlations in their amplitude modulation, as measured by the DFA exponent in (E, F). Theoretically, a DFA exponent is 0.5 for random signals and >0.5 for signals with temporal correlations; however, finite signal duration leads to variation in DFA exponents, making an exponent <0.6 a more reliable indicator of random fluctuations in real-world data. A critical network (*green circle*) shows presence of strong LRTC, as quantified by the DFA exponent approaching 1, computed on the amplitude envelope of alpha oscillations (indicated by the*red*line in B). This contrasts with sub-critical or super-critical networks (*blue*or*red*, respectively), which show lower DFA exponents, and to white-noise signals (*black*) with the DFA exponent of <0.6, indicating the absence of LRTC. (G) The combination of oscillations amplitude and DFA exponent can be used to infer the E/I balance of networks. (H) Normalized fluctuation function, log_10_ <*nF*(*t*)>, computed for a window size of 5 seconds, is a good proxy of the DFA exponent of the network (Pearson correlation, ***p< 0.001). Each value represents the average of 10 networks for each combination of excitatory and inhibitory connectivity percentage, where networks were filtered based on the criticality of avalanches,*k*, between 0.8 and 1.2. (I) Joint fluctuations in the amplitude and scaling of oscillations enable estimation of excitation-inhibition ratio of a neuronal network. Correlation between the windowed amplitude and*nF*(*t*) values is used to estimate a functional E/I ratio (*fE/I*), which is <1 for sub-critical networks, >1 for super-critical networks, and equal 1 for critical networks. (A) reprinted fromAvramiea et al. (2020), Copyright CC BY 4.0. (G) reprinted fromBruining et al. (2020).

Detrended fluctuation analysis (DFA) exponent is a measure of the temporal structure, which is based on the mean fluctuation function, that is, the mean variability in a signal, over a number of different time scales (Hardstone et al., 2012). It estimates the power-law decay of long-range temporal correlation (LRTC) within the signal. LRTC is a robust empirical feature of critical-state oscillations (Linkenkaer-Hansen et al., 2001,^2007^;Monto et al., 2007) and is dependent on the E/I balance. It estimates the temporal structure of oscillation amplitude and reflects the level of criticality in the network. The mean fluctuation of the signal is computed as a function of growing time-window sizes,*F(t)*, and the relationship is plotted in log-log coordinates (Fig. 1E). The slope of the line between the log of the fluctuation and the log of the window sizes fitted in the time scales of interest is the DFA exponent and provides the scaling of the decay of LRTC. The DFA exponent serves as a proxy of the Hurst scaling parameter (Hurst, 1951;Mandelbrot & Wallis, 1969) and gauges the correlation of fluctuations at different time lags. The DFA value of 0.5 indicates an uncorrelated random signal (i.e., absence of LRTC), whereas the value >0.5 indicates the presence of positive auto-correlations and their strength. DFA is performed on the amplitude envelope of the bandpass-filtered signal. The main steps are detailed inSupplementary Methods S1.

### 2.2 *fE/I* algorithm

The algorithm was developed based on an extended version of the Critical Oscillations (CROS) computational model of neuronal oscillations (Poil et al., 2012) which mimics the signals observed in human M/EEG recordings (Fig. 1A–F). In*fE/I*, the E/I ratio is estimated from the windowed covariation of the average amplitude and amplitude modulation (the temporal auto-correlation structure) of frequency-specific activity, in the presence of significant LRTC in the signal (Fig. 1G–I). LRTC are assessed via a DFA exponent (Section 2.1) in the time scales of interest (Fig. 1E–F). Taken alone, the DFA exponent cannot distinguish sub- from super-critical activity (Fig. 1G). Neither can the amplitude of oscillations, which changes monotonously with E/I balance, tell where the network is critical. The combination of the two, on the other hand, can be used to tell apart sub-, critical, and super-critical dynamics (Fig. 1H–I). The correlation of amplitude and DFA is positive for a network operation in a slightly sub-critical state (Fig. 1I,*left*), zero in a critical state (Fig. 1I,*middle*), and negative in a slightly super-critical state (Fig. 1I,*right*). Given that networks operating in these regimes exhibit co-variation in amplitude and temporal structure, we can use a sliding-window approach to quantify this co-variation and, thus, infer the E/I balance of the underlying networks. Of note, when networks are far from criticality as reflected in DFA exponents <0.6, there is no co-variation between amplitude and temporal structure and, therefore, the DFA exponent of 0.6 is used as a threshold to compute the*fE/I*ratio (Bruining et al., 2020).

Given the presence of LRTC in the signal reflected by the DFA exponent greater than 0.6,*fE/I*is then computed by correlating the amplitude and LRTC in short windows (Supplementary Methods S2.2). Windowed LRTC, in this case, is estimated through the normalized fluctuation function, which serves as a reliable proxy of the DFA exponent on short time scales (Fig. 1H). Values of the fluctuation function,*log_10_< F(t)>*, for a window size scale proportionally with both the amplitude of the signal and LRTC (Bruining et al., 2020). So, doubling the amplitude does not change the slope of the fluctuation function (seefigure 2CinBruining et al., 2020), and we can reliably approximate the DFA exponent from one window size by removing the influence of amplitude on*F*(*t*) for that window size. The main steps of the*fE/I*algorithm are detailed inSupplementary Methods S2.1.

**Fig. 2.**
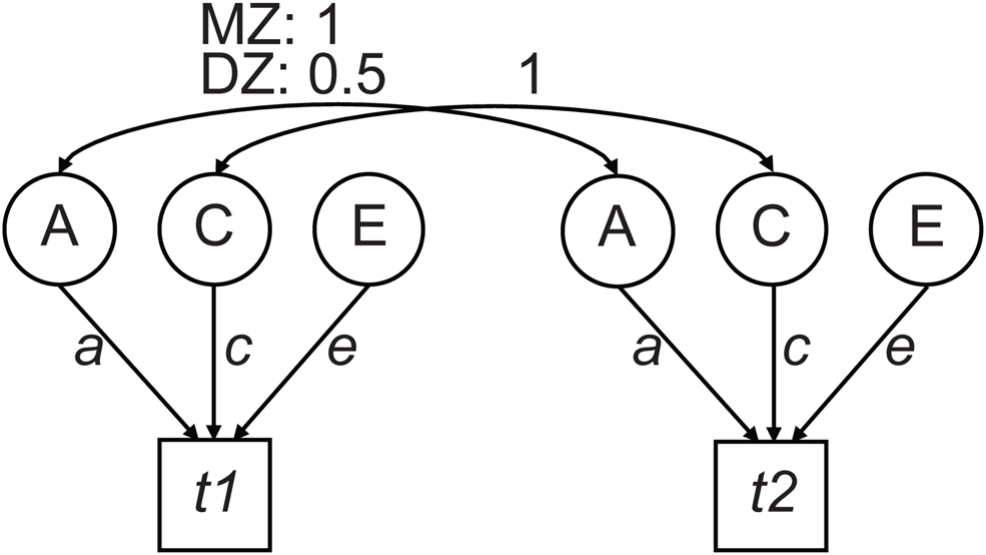
Variance decomposition. Path model for estimating additive genetic effects (A), common environmental effects (C), and unique environmental effects (E) on the observed variables for twins 1 and 2 (*t1*,*t2*, respectively). Factor C correlates 1 between twins as this reflects the environmental effects shared between the twins (such as the shared rearing environment). Factor E is non-shared, unique variation and correlates zero between the twins (no arrow). Genetic factor A correlates depending on the genetic overlap, that is, 1 for MZ twins, and 0.5 for DZ twins, and reflects the influence of additive genetic effects. The relative contribution of factors A, C, and E is determined by path loadings*a*,*c*and*e*.

### 2.3 Optimizing *fE/I* for application in narrow frequency ranges

The primary step in both DFA and*fE/I*is bandpass finite-impulse-response (FIR) filtering which extracts the frequency-specific activity from the signal. To extend the application of*fE/I*(and DFA) across the frequency spectrum, we first optimized the resolution of frequency binning in the range of 1–150 Hz by examining the frequency response of FIR filters on simulated white-noise data (Supplementary Methods S3.1). Next, we examined how the filtering affected the estimation of LRTC in the activity in the defined frequency bins and adapted the lower end of the DFA fitting interval in a frequency-dependent manner (Supplementary Methods S3.2). We further validated these settings by computing spectral*fE/I*in the signals from the CRitical OScillations (CROS) computational model (Section 3.1) and in human EEG data (Sections 3.2–3.3).

### 2.4 Networks and signals from the CRitical OScillations (CROS) model

#### 2.4.1 Overview

In our study, we used the CRitical OScillations model, extensively described in previous papers (Avramiea et al., 2020;Bruining et al., 2020;Poil et al., 2012). Specifically, we employed the adapted version of the model published inAvramiea et al. (2020), and details, including equations and model parameters, are provided inSupplementary Methods S4. In the CROS model, a network signal is created by summing the total number of neurons spiking at each time-step with a Gaussian white noise signal of the same length with mean = 0 and standard deviation = 3. This level of white noise is set to allow all networks to achieve a time-varying phase, which is not the case without adding the noise, when there are silent periods in the network (Avramiea et al., 2020).

#### 2.4.2 Neuronal avalanches

A neuronal avalanche is defined as a period where neurons are spiking above a certain threshold. In our case, it is set to half of the median activity. The size of the avalanche is the number of spikes during this period. The*k*index (Poil et al., 2012;Shew et al., 2009) is determined as the difference between the distribution of our data and a power-law, by calculating the average difference of the cumulative distribution of a power-law function,*P*, (with exponent -1.5 for size and -2.0 for the duration) and that of our experimental data,*A*, at 10 equally spaced points on a logarithmic axis (β) and adding 1.

$$k=\frac{1}{10}\sum_{i=1}^{10}\left(P\left(\beta_{i}\right)-A\left(\beta_{i}\right)\right)+1$$

A sub-critical distribution is characterized byk<1, and a super-critical distribution byk>1, whereask=1indicates a critical network.

#### 2.4.3 Validation sample

To validate modifications in DFA and*fE/I*across the frequency spectrum, we sampled networks from the CROS phase space to cover a range of sub-critical, critical, and super-critical regimes. A sample was obtained by setting the excitatory*C_E_*and inhibitory*C_I_*connectivity parameters from 30% to 42% and 50% to 38%, respectively, at 0.5% intervals. This resulted in 25 parameter combinations of excitatory/inhibitory connectivity along a diagonal orthogonal to the critical line, that is, the sum of excitatory and inhibitory connectivity was constant and equal 80%. We ran each combination 20 times, each leading to a slightly different network initialization due to the probabilistic nature of connectivity. For each combination, this resulted in 20 signals of 1,000 seconds with a sampling frequency of 1,000 Hz. The avalanche size metrics,*k*index, was used as the ground truth of the degree of the overall network’s criticality.

#### 2.4.4 Phase-space plots

Networks were also sampled from the CROS phase space by varying the excitatory and inhibitory connectivity parameters from 20% to 100% each in steps of 2%. This was done to showcase comprehensive phase-space plots of LRTC and*fE/I*across the entire range of parameter combinations (*n*= 1,681), producing varying levels of critical dynamics. Each combination was iterated 10 times, producing 10 signals, each lasting 1,000 seconds and sampled at a frequency of 1,000 Hz.

#### 2.4.5 DFA and fE/I analyses

The upper bound of the DFA fitting interval was fixed at 30 seconds. For the lower bound, we used the values obtained during optimization (Supplementary Methods S3.2). The amplitude envelope of the filtered signal was extracted via Hilbert transform using the*MNE*function*apply_hilbert*. The window size of 5 seconds was used to compute*fE/I*(Supplementary Methods S2.2).

### 2.5 Empirical data

#### 2.5.1 Twin dataset

To substantiate a biological relevance of computing*fE/I*across the spectrum, we determined its genetic basis using the classical twin design (Boomsma et al., 2002). Structural equation models for estimating heritability were applied to an EEG dataset recorded in twins from the Netherlands Twin Register (NTR). We used the dataset previously inLinkenkaer-Hansen et al. (2007)where a strong genetic contribution to long-range temporal correlations (as estimated with DFA) was found in alpha and beta oscillations.

##### 2.5.1.1 Data overview

The dataset comprised 368 subjects, of which 80 monozygotic (MZ) and 104 dizygotic (DZ) twin pairs (194 females, 16.5–19.5 years). The EEG data included resting-state eyes-closed recordings of 3–6 minutes with 14 electrodes (10–20 system) sampled at 250 Hz. The recordings were bandpass-filtered at 1–35 Hz and cleaned from artifacts in the EEGLAB toolbox. The details of preprocessing are described in Materials and Methods ofLinkenkaer-Hansen et al. (2007). We further imported the files into*Python*using*MNE*(Gramfort et al., 2013).

##### 2.5.1.2 Analysis of EEG data

DFA and*fE/I*were computed in the cleaned signals across narrow-range frequencies in the range of 1–28 Hz. We used the frequency binning approach obtained inSupplementary Methods S3.1. Adaptive fitting was used across frequencies, in which we optimized the lower bound of the DFA fitting interval (Supplementary Fig. S3M,*orange*line). The upper bound was fixed at 30 seconds. The window size of 5 seconds was used to compute*fE/I*. On average, 31% of electrodes (SD = ± 11%) had DFA < 0.6 (and, thus, missing*fE/I*) across frequencies. First, we calculated the average percentage of electrodes with DFA < 0.6 across subjects for each frequency bin and then averaged across frequency bins.

##### 2.5.1.3 Variance decomposition and heritability

In the classical twin design, contributions of genetic and nongenetic (i.e., environmental) effects to the total variance are modeled by comparing monozygotic and dizygotic twin covariance. Additive genetic (A), common environmental (C), and unique environmental (E) variance components were estimated as depicted in the path model (Fig. 2). We used structural equation models with maximum-likelihood estimation implemented in the*OpenMx*software package (Boker et al., 2023;Neale et al., 2016) in*RStudio*(Posit team, 2023) using*R*version 4.3.2 (R Core Team, 2023) to derive the model parameters (*a*,*c*, and*e*) with sex and age included as covariates. Likelihood ratio tests were conducted to evaluate the significance of individual components: twice the difference in likelihood between the full model and a model with a parameter fixed to zero is approximately chi-square distributed with the number of dropped parameters as degrees of freedom. Significance level was set at 0.05. Heritability was estimated as the fraction of the variance that was genetic*a*^2^/ (*a*^2^+*c*^2^+*e*^2^). In cases where the contribution of factor C was nonsignificant, heritability was simplified to*a*^2^/ (*a*^2^+*e*^2^). Model fitting was conducted per channel and frequency bin.

#### 2.5.2 Resting-state EEG

##### 2.5.2.1 Data overview

We used a publicly available EEG dataset which is part of the MPI Leipzig Mind-Brain-Body database (LEMON) (Babayan et al., 2019). Cleaned resting-state EEG recordings of 16 minutes, 8 minutes of which with eyes closed (EC) and 8 minutes with eyes open (EO), were available in 200 healthy participants (146 females, age range 22.5–77.5 years). Recordings were obtained with a BrainAmp MR plus amplifier using 62-channel active ActiCAP electrodes (61 scalp electrodes + 1 VEOG electrode; 10-20 extended localization system) and referenced to FCz.

##### 2.5.2.2 Preprocessing

The data were downsampled from 2,500 Hz to 250 Hz and bandpass-filtered within 1–45 Hz (8th order Butterworth filter). Bad channels and transient artifacts were manually identified and removed. On average, 0.93 channels were removed (SD = ± 1.2). Eye blinks, eye movements, and heartbeat artifacts were further removed via independent component analysis preceded by principal-component dimensionality reduction in EEGLAB (Delorme & Makeig, 2004; see methods inBabayan et al., 2019).

##### 2.5.2.3 Analysis of EEG data

DFA and*fE/I*were computed in the cleaned signals across narrow-range frequencies in the range of 1–45 Hz. We used the frequency binning approach explained inSupplementary Methods S3.1. Adaptive fitting was used across frequencies (Supplementary Fig. S3M,*orange*line). The upper bound was fixed at 30 seconds. The window size of 5 seconds was used to compute*fE/I*. On average, 31% of channels (SD = ± 16%) had DFA < 0.6 in eyes-open rest across frequencies, and 18% (SD = ± 14%) had DFA < 0.6 in eyes-closed rest across frequencies. First, we calculated the average percentage of channels with DFA < 0.6 across subjects per frequency bin and then averaged across frequency bins.

##### 2.5.2.4 Statistical analysis

We employed linear mixed-effects models (LMMs) to compare*fE/I*between EO and EC conditions. Condition, age, and their interaction were specified as fixed effects. To account for the paired nature of the data, we included a random intercept for each subject. At the whole-brain level, LMM was fitted using restricted maximum likelihood per frequency bin, and Bonferroni correction accounted for multiple comparisons across bins. At the electrode level, LMM was fitted for two frequency bins (1 alpha and 1 beta), identified as significant at the whole-brain level. Bonferroni correction addressed multiple comparisons across 61 electrodes. Significance was set at 0.05. LMMs and multiple comparison correction were implemented using*MixedLM*and*multipletests*from the*statsmodels Python*package (Seabold & Perktold, 2010), respectively.

## 3 Results

To explore the possibility of assessing functional E/I in multiple narrow frequency bands, we optimized the spectral and temporal characteristics of the bandpass filters (Supplementary Methods S3.1 and S3.2) and evaluated*fE/I*on the CROS model and in human EEG.

### 3.1 E/I ratio can be reliably measured by *fE/I* from near-critical oscillations across frequencies

After having characterized the spectral and temporal width of the bandpass filters, we validated the*fE/I*algorithm across frequencies on the signals from the CROS model, which can be tuned in and out of the near-critical regime by changing the E/I ratio of synaptic connections (Poil et al., 2012). We define the near-critical state by DFA > 0.6, supported by the avalanche size criticality*k*(Section 2.4,*Neuronal avalanches)*in the range of 0.87–1.14 which was determined from the CROS phase space (Section 2.4,*Phase-space plots*). Thus, when referring to sub- or super-critical dynamics with DFA > 0.6, we encompass them as being in the near-critical state.

**Fig. 3.**
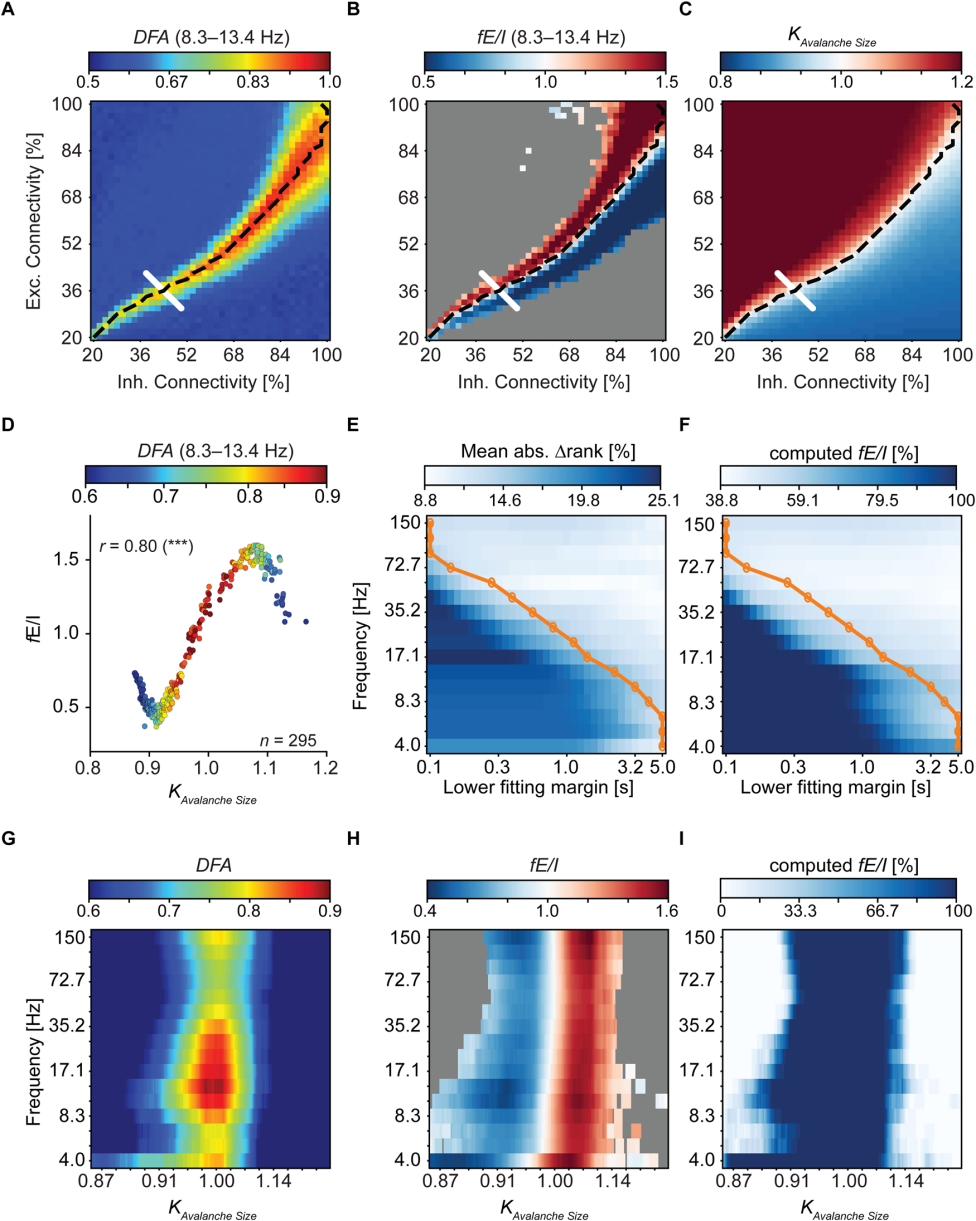
DFA and*fE/I*reveal near-critical oscillations across frequencies. (A–C) DFA,*fE/I*as well as*k*criticality values are shown. Each network in the phase space depicts the mean DFA,*fE/I*, or*k*across 10 network’s realizations. The mean DFA and*fE/I*were averaged across the two alpha frequency bins.*fE/I*was computed only when the mean of the network’s DFA across all its realizations was >0.6, and the networks for which*fE/I*was not computed are shown in*gray*.*Dashed black*line indicates critical neuronal avalanches.*White*line indicates sampled networks, crossing the critical range of the CROS phase space. Higher resolution*white*line (0.5% intervals) was interpolated onto the lower-resolution grid (2% intervals). (D)*fE/I*and*k*exhibit a strong correlation with each other (Spearman’s rank correlation). (E, F)*fE/I*accuracy measured by the mean absolute rank difference, ∆rank, and the percentage of networks for which*fE/I*was computed are plotted against the lower window of DFA fitting interval, respectively. (G, H) Critical oscillatory activity across a broad range of frequencies exhibits long-range temporal correlations and certain E/I ratio emerging in networks operating in or close to the critical state. Networks were arranged on the x-axis based on their corresponding*k*values, and a moving average was calculated using a window size of 20 and an overlap of 80%. Both-side padding was applied using the respective first and last values. (I) Networks deviating farther from the critical state result in a reduced number of eligible cases for the*fE/I*algorithm. Both-side padding with*NaN*s was applied for the number of computed*fE/I*values before computing the moving average. ***p< 0.001.

We sampled 25 networks with varying combinations of excitatory and inhibitory connectivity parameters along a diagonal orthogonal to the critical line in the CROS phase space (Section 2.4,*Validation sample*) (Fig. 3A–C,*white*line). Each combination was realized multiple times (*n = *20) to account for variations in network topology and the spreading of avalanche-like activity. E/I balance and criticality of avalanches, estimated with*fE/I*(Section 2.2) and*k*index, respectively, exhibited a strong correlation with each other (Spearman’s*r*= 0.80,*p*<< 0.001), shown for the alpha frequency range inFigure 3D. We used*k*as a benchmark of criticality for*fE/I*across the frequency spectrum, aiming to illustrate the impact of LRTC overestimation due to correlations introduced by the filter at shorter time scales and the incorporation of signals with overestimated DFA exponents on*fE/I*accuracy.

We order-matched*fE/I*with*k*at each time scale of the DFA fitting interval and calculated the average absolute rank difference between*fE/I*and*k*per frequency component across all CROS signals (*n*= 25 x 20). The ranks of*fE/I*and*k*were normalized by the number of signals with computed*fE/I*values. This provided an estimation of how well*fE/I*reflected the level of criticality across the diagonal orthogonal to the critical line. A mean absolute rank of 0% corresponds to a perfect agreement between*fE/I*and*k*, while a value of 33% suggests an agreement at chance levels. At shorter time scales, DFA artificially exceeds the threshold of 0.6, and*fE/I*is computed for signals lacking temporal structure, evident in the increased percentage of computed*fE/I*values (Fig. 3F,*dark blue*region). The algorithm does not perform well for these signals, which results in reduced overall accuracy of*fE/I*, reflected in the rise in the mean absolute rank difference at time scales affected by the filter bias (Fig. 3E,*dark blue*region). Using the optimized time scales for fitting DFA across the frequency spectrum (Fig. 3E,*orange*line), detailed inSupplementary Methods S3.2, yielded the mean absolute rank difference of 13% on average across frequencies, where the values ranged from 10% to 16%. Concurrently, the percentage of computed*fE/I*values varied from 44% to 73%, with a mean of 54%.

Notably, we observed that near-critical oscillations, as operationalized in CROS, emerged in narrow-range frequencies across the entire spectrum (Fig. 3G–I). DFA peaked for critical networks (Fig. 3G) and showed the widest transition between sub- and super-critical dynamics in the delta, alpha, and low-beta frequencies.*fE/I*distinguished between inhibition-dominated (i.e., sub-critical) and excitation-dominated (i.e., super-critical) activity in all frequencies and showed that a certain balance of excitation and inhibition was achieved for critical oscillations to emerge (Fig. 3H). Generally, the*fE/I*algorithm in each frequency was applicable to networks within the respective range of transition between sub- and super-critical dynamics with detectable temporal correlations (i.e., DFA > 0.6,Fig. 3G). This was reflected by the percentage of computed*fE/I*surging in the critical range and gradually dropping to 0% when moving away from criticality, where there were less and less networks with temporal correlations satisfying the DFA threshold of the*fE/I*algorithm (Fig. 3I).

### 3.2 *fE/I* is heritable across the spectrum

**Fig. 4.**
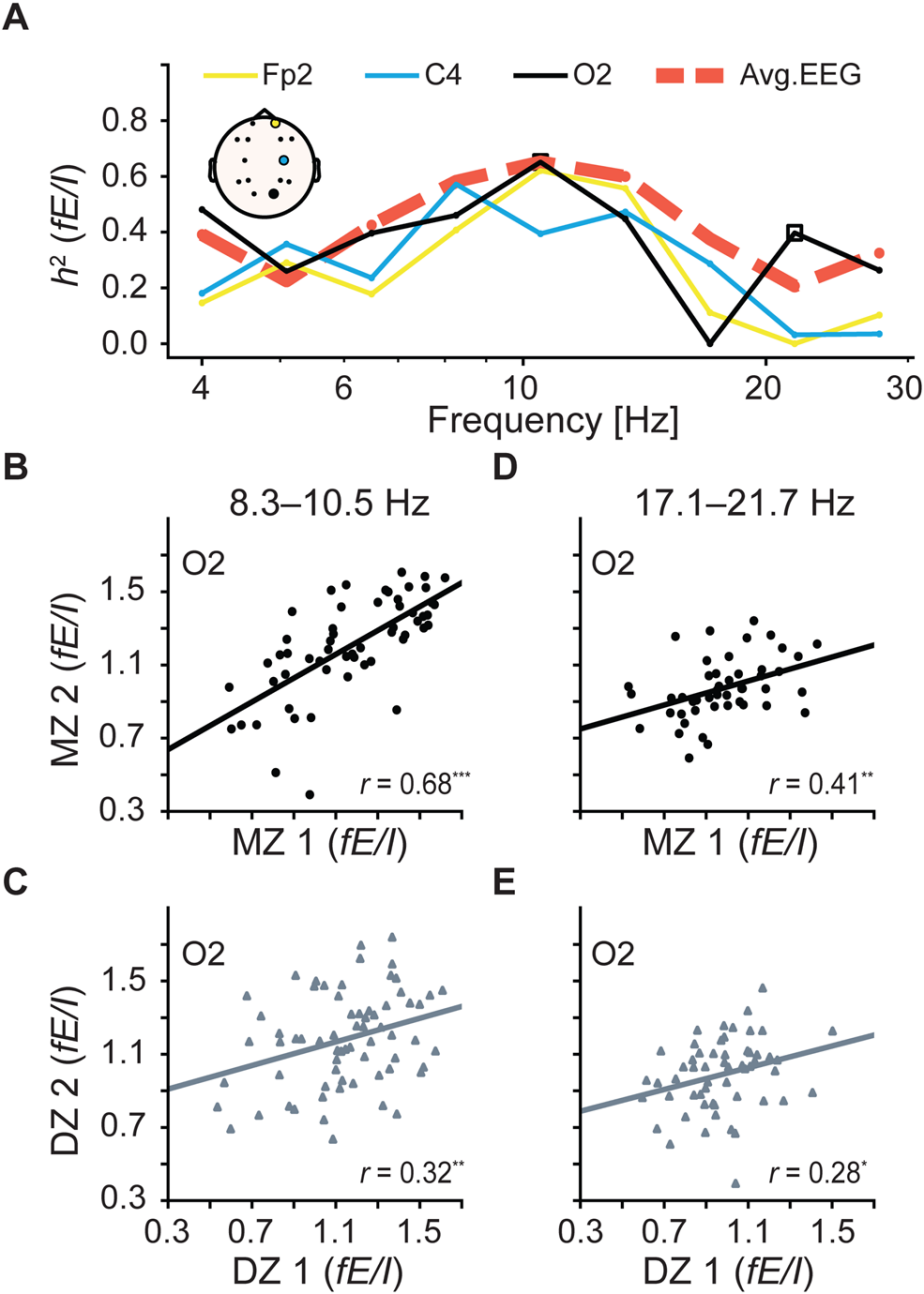
*fE/I*is heritable across frequencies. (A) Heritability,*h^2^*, of whole-brain*fE/I*is shown with traces for electrode-level*h^2^*of 3 EEG channels included. (B–E) Correlations (*r*) between monozygotic (MZ) twins (*circles*) and between dizygotic (DZ) twins (*triangles*). The MZ versus DZ correlations at the O2 electrode are respectively shown for (B, C) alpha and (D, E) beta frequency bins, indicated with hollow squares in A. In the scatterplots, the significance of correlations is indicated with asterisks, where * signifies*p*< 0.05, **p< 0.01, and ***p < 0.001.

The results from the model underscore that*fE/I*may be used as a versatile indicator of near-critical network activity across frequencies, making it suitable for probing E/I balance in a broad range of neural activities. To further enhance our understanding of the functional role of E/I regulation in ongoing oscillations across the spectrum, we explored the biological significance of*fE/I*within distinct frequency components using empirical resting-state EEG data from monozygotic (*n = *80) and dizygotic (*n = *104) twins (Section 2.5.1). We found that heritability of*fE/I*was highest in the alpha frequency at 8–10 Hz, ranging from 39% to 66% across electrodes (Fig. 4A, shown for 3 electrodes;Supplementary Table S1). Similar results were observed at 6.5–8 Hz and 10.5–13 Hz, where heritability across electrodes ranged from 33% to 57% and from 34% to 64%, respectively. Notably, in the alpha frequencies (6.5–13.4 Hz), all electrodes showed a significant genetic contribution to*fE/I*. Zooming in on one of the occipital electrodes where alpha oscillations are usually the strongest, we observed that monozygotic correlations of*fE/I*in the alpha bins were twice as strong as dizygotic correlations (Pearson*r*(MZ) = 0.68,*p*< 0.001 and*r*(DZ) = 0.32,*p*< 0.01 for O2 electrode at 8–10.5 Hz inFig. 4B and C, respectively). Higher similarity between MZ twins compared to DZ twins suggests a genetic contribution. DZ twins share about 50% of their genetic material, so their correlation provides a baseline for what would be expected due to genetic influence if it were lower than that of MZ twins. For example, for O2 electrode, through structural modeling, we estimated the total variance in*fE/I*to be 0.069, with 65% of this variance explained by genetic factors (*p << *0.001). This pattern was generally consistent across electrodes (Supplementary Table S1). In other frequencies, heritability was highly variable across leads. It ranged from 0% to 62% (median = 27%) in frequencies covering the range of 1–6.5 Hz and from 0% to 42% (median = 15%) in the beta bins at 13–28 Hz (Supplementary Table S1). In electrodes with significant effects (*p*< 0.01), heritability ranged from 37% to 62% at 1–6.5 Hz, and from 33% to 42% at 13–28 Hz (Fig. 4D and E, shown for O2 electrode at 17–22 Hz).

Interestingly, heritability estimates of the whole-brain average*fE/I*were significant in 6 out of 9 frequency bins (Supplementary Table S1), ranging from 32% to 65%, with a peak in the alpha frequencies. The average*fE/I*across all electrodes reflected near-critical brain dynamics operating between inhibition-dominated and E/I balanced regimes in delta, theta, and beta oscillations (0.90 ± 0.10, 0.94 ± 0.10, 0.91 ± 0.12, 0.89 ± 0.12, 0.92 ± 0.11, and 89 ± 0.09 at 1–4 Hz, 4–5 Hz, 5–6.5 Hz, 13–17 Hz, 17–22 Hz, and 22–28 Hz, respectively,Supplementary Table S1). In the alpha bins,*fE/I*indicated dynamics fluctuating at criticality with balanced excitatory and inhibitory forces.

### 3.3 *fE/I* detects narrow-range reductions in E/I ratios during eye-opening

**Fig. 5.**
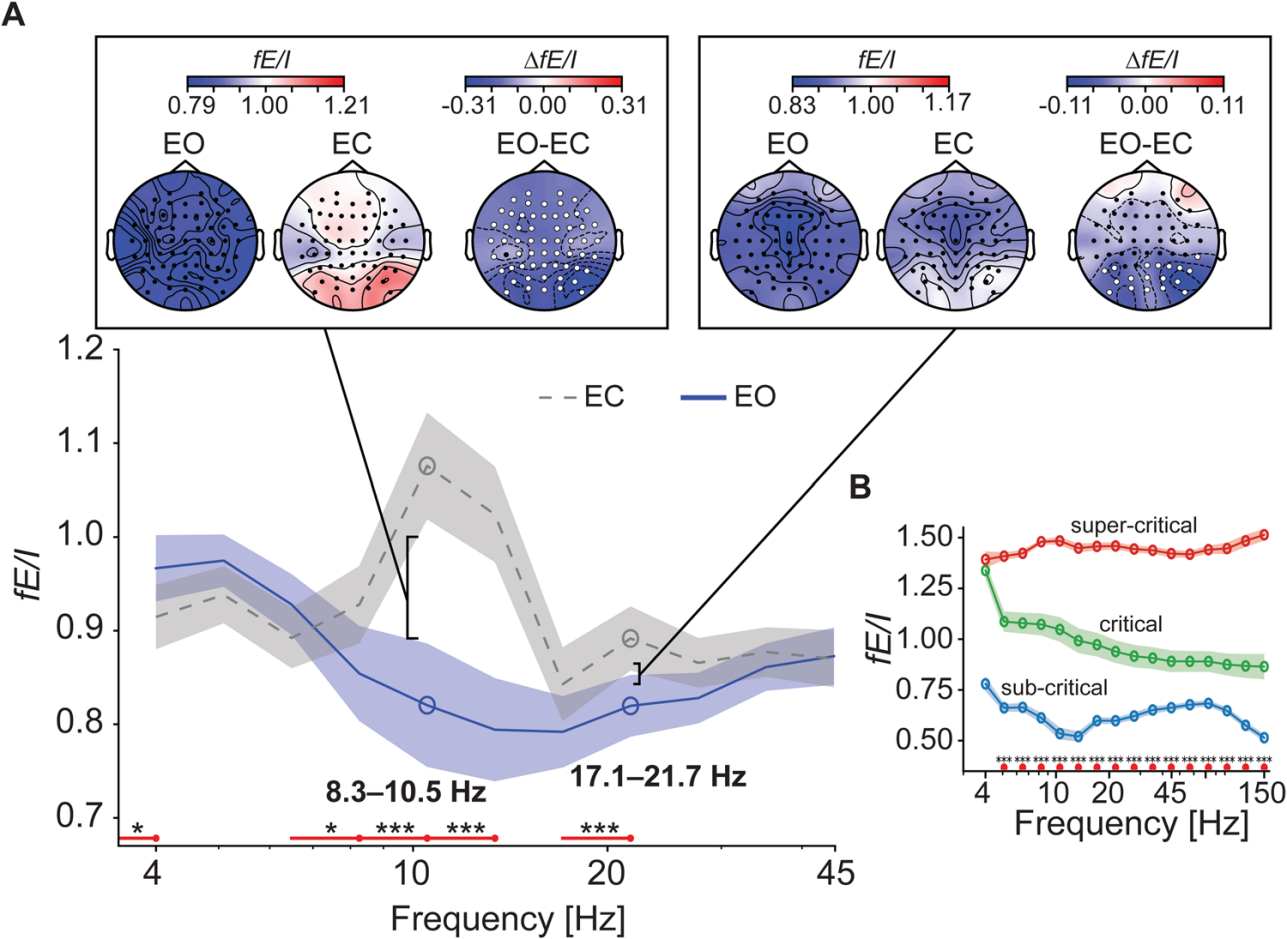
*fE/I*detects reductions in E/I ratios in restricted frequency ranges and anatomical locations during eye-opening. (A) The*gray*and*blue*curves in the main plot represent LMM-estimated brain-wide*fE/I*for EC (intercept) and EO (condition effect), respectively, with shaded areas depicting 95% confidence intervals. Significant frequency bins are highlighted in*red*, reflecting corrected*p*-values (Bonferroni, across 11 frequency bins) for the condition effect after running LMM. Topographies in the insets reflect the electrode-level means across individual subject*fE/I*s for EC and EO, as well as the mean differences between EO and EC. White circles on the topographies of the mean differences (*EO-EC*) indicate significant electrodes based on corrected*p*-values (Bonferroni, across 61 electrodes) for the condition effect after running LMM. (B) The curves represent the average*fE/I*across 20 runs of each network, with shaded areas indicating SEM. The networks––sub-critical (*red*), critical (*green*), and super-critical (*blue*)––were selected from the diagonal orthogonal to the critical line. Significant frequency bins are shown in*red*, reflecting corrected*p*-values (Bonferroni, across 16 frequency bins) of the condition variable of the ANOVA test. Asterisks indicate significance levels: *p< 0.05, **p< 0.01, and ***p < 0.001.

The model showed that*fE/I*can reliably infer E/I ratio from oscillations across a broad range of frequencies. Here, we examined the sensitivity of*fE/I*to discern frequency-specific changes in real neural signals, across brain states. We analyzed resting-state EEG data from healthy subjects (*n = *200), recorded during both eyes-open and eyes-closed rest conditions (Section 2.5.2). We fitted LMMs to estimate the effects of condition, age, and the interaction between age and condition on*fE/I*. We found that whole-brain*fE/I*was significantly decreased in multiple narrow-range frequencies during eyes-open rest compared to eyes-closed rest (Fig. 5A). The effect was observed in the alpha range between 6.5–13 Hz, with the peak at 8–10 Hz (coefficient = -0.26, 95%,*p*<< 0.001), and in the beta range between 17–22 Hz (coefficient = -0.07,*p*= 0.0002) (Fig. 5A). In the delta range between 1–4 Hz,*fE/I*increased in eyes-open rest (coefficient = 0.05,*p*= 0.04) (Fig. 5A). In the upper-alpha frequency bin between 10.5–13 Hz,*fE/I*decreased with age in eyes-closed rest with a coefficient of -0.002 (*p*= 0.01), indicating a decrease in*fE/I*of 0.02 per decade of aging. Conversely,*fE/I*increased with age in the eyes-open rest condition (coefficient = 0.003,*p*= 0.0001), indicating an increase of 0.03 per decade of aging. These findings suggest that the difference in*fE/I*between eyes-open and eyes-closed rest diminishes with age. Similar results were found between 8–10 Hz, where*fE/I*in eyes-open rest increased with age (coefficient = 0.002,*p*= 0.03), while it decreased in eyes-closed rest (coefficient = -0.002,*p*= 0.06). The latter effect, however, was nonsignificant after Bonferroni correction.

The reduction in alpha*fE/I*was spatially widespread across the brain (Fig. 5A,*left*inset), indicating a pervasive shift towards inhibition-dominated dynamics during eye-opening. In the beta band, the impact was primarily localized in the parietal electrodes (Fig. 5A,*right*inset). Taken together, these findings suggest that*fE/I*is sensitive to alterations in frequency-specific neural activities, underscoring the significance of assessing the E/I ratio within narrow frequency ranges. Importantly, this could not be corroborated in the CROS model, where activity in different frequencies stems from the same underlying circuit. In the model,*fE/I*successfully distinguishes between networks operating in inhibition-dominated (*n = *20), excitation-dominated (*n = *20), and E/I-balanced (*n = *20) regimes across the entire frequency spectrum (Fig. 5B).

## 4 Discussion

To optimize the application of the*fE/I*algorithm within narrow frequency ranges across the spectrum, we analyzed the temporal and spectral characteristics of the underlying filters. This analysis enabled us to identify optimal resolution for spectral binning and determine the shortest time scales needed to assess auto-correlations in spectrally-refined oscillatory activity. We validated the newly established parameters using the CROS model, confirming that*fE/I*discriminates E/I connectivity differences across a wide range of frequencies. We also established a genetic basis of resting-state*fE/I*across the frequency spectrum. Finally, we demonstrated that eye-opening had a spectrally and anatomically targeted impact on*fE/I*, revealing reductions in distinct frequency components and brain regions.

### 4.1 Advantages of the narrow-band frequency approach

Previous studies have shown the value of a data-driven hypothesis-free approach to investigate the spectral nature of pathological brain activity. In contrast to canonically-defined frequency bands, using finer frequency resolution allowed to uncover similar patterns between adjacent frequency bins and derive frequency-band boundaries based on the observed effects (Fuscà et al., 2023;Houtman et al., 2021;Stuiver et al., 2024;S. H. Wang et al., 2024). Recently, the*fE/I*algorithm has also been applied to higher frequencies in MEG and LFP recordings (Javed et al., 2023;Kat et al., 2024), which supports the relevance of validating*fE/I*across the spectrum.

Here, we defined the resolution for frequency binning in the range between 1–150 Hz, encompassing higher gamma frequencies, which are of growing interest in research. Alterations in high-frequency gamma activity, for example in Alzheimer’s disease, have been linked to synaptic dysfunction and disruptions in network E/I balance (Hijazi et al., 2020,^2023^;Verret et al., 2012). A wider frequency range also provides a means for translational MEG/ECoG/LFP studies between human and other species, such as mice, where frequencies relevant for behavior or disease can exceed 100 Hz (Buzsáki, 2006). We generated 16 frequency bins—one between 1–4 Hz and 15 on a log scale within 4–150 Hz. Such bin allocation was driven by the observed overlap between the power spectra of the adjacent bins (Supplementary Methods S3.1).

Compared to the five commonly used traditional frequency bands, our approach provides a more detailed and granular representation of the frequency spectrum, facilitating a finer examination of neural oscillations across the spectrum. This design also circumvents redundancy seen in denser methods. The use of 1-Hz-wide bins becomes impractical for broad frequency ranges of interest. It introduces redundant bins at higher frequencies and penalizes results with an increased number of comparisons when performing multiple comparison correction analyses. For example, in the Bonferroni correction, one would have to correct across 149 frequency bins instead of 16, which reduces statistical power and the likelihood of detecting smaller effects that may be present.

### 4.2 Using CROS to reveal near-critical activity across a wide range of narrow frequency bands

The CROS model consists of connected integrate-and-fire neurons, whose spread of activity manifests, at the population level, as network oscillations, which allows to link the scaling behavior of avalanches of spiking activity and of amplitude fluctuations in network oscillations (Poil et al., 2012). CROS was shown to produce narrow-band oscillations in the alpha and beta bands (Poil et al., 2012) (Fig. 1C). However, the relationship between the criticality of oscillations and the balanced excitation and inhibition has been previously studied in the model only in the alpha band (Poil et al., 2012) (Fig. 1B–F). Here, we show that the CROS model also generates critical activity fluctuations across a wide range of narrow frequency bands, spanning from 1–4 Hz to 118–150 Hz (Fig. 3G–H), even though no clear oscillatory peaks are present in the power spectrum at the higher frequencies (Fig. 1C). This is similar to empirical EEG/MEG, which exhibit scale-free dynamics across a wide range of frequencies (Linkenkaer-Hansen et al., 2001;Monto et al., 2007) even though it rarely has clear peaks in the gamma ranges. In contrast, white noise shows no features of criticality across frequencies (Supplementary Fig. S3I, M, N). The ability of the CROS model to replicate important features of population dynamics observed empirically in EEG/MEG studies makes it a valuable tool in studying the application of the*fE/I*algorithm to both periodic and aperiodic activity. Unlike in CROS, however, signal mixing contaminates sensor data in EEG/MEG, which has been shown to affect DFA (Blythe et al., 2014) and, therefore, inevitably also*fE/I*quantifications. Thus, we recommend source modeling whenever high-density EEG/MEG is available.

### 4.3 Including shorter time scales in DFA fitting for handling missing values in *fE/I*

The application of the*fE/I*algorithm is limited to networks exhibiting near-critical dynamics, as indicated by significant long-range temporal correlations, which for practical reasons is determined as a DFA exponent >0.6 (Bruining et al., 2020). In cases of sub- or super-critical networks deviating significantly from criticality, the algorithm yields a missing*fE/I*value. This is because in the absence of long-range temporal correlations, there is no co-variation between amplitude and the fluctuation function (see the grey regions ofFig. 1G). Importantly, this limitation is methodological and does not imply the absence of a specific E/I ratio in the network. In pathologies with strong E/I imbalances, the algorithm may not be applicable, albeit this is too early to say. This principally depends on the robustness of LRTC in these signals, which, in turn, may be influenced by the signal-to-noise ratio of the signal, the signal’s length, or artifacts (Hardstone et al., 2012). In cases where DFA exponents in real EEG data are <0.6, we recommend inspecting each signal and plotting its power spectrum and the fluctuation function used to compute the DFA exponent. It may indicate whether this is a robust result, or the factors mentioned above might be involved.

While DFA thresholding is necessary for accurate*fE/I*computation, we show that LRTC estimation reliability and sensitivity can be enhanced by incorporating shorter time windows into the fitting interval as there are many more windows in the low time scale (Supplementary Methods S3.2). This modification allowed for the identification of weak LRTC and accurate computation of*fE/I*in CROS networks with DFA values approaching the threshold of 0.6. This may be especially important for higher frequencies where absolute time scales can be considered relatively longer than those of lower-frequency oscillations if the time is measured in oscillation cycles. Overall, this adjustment addressed the challenge of missing values in the*fE/I*algorithm, with particularly prominent improvements observed in networks in the sub-critical regime (Supplementary Fig. S4). This is important, considering the documented benefits of sub-critical dynamics in the brain (Avramiea et al., 2020;Fagerholm et al., 2015;Irrmischer et al., 2018;Priesemann et al., 2014) and the implications of pathological inhibition-dominated dynamics in brain disorders (Dani et al., 2005;Fernandez & Garner, 2007;Houtman et al., 2021;Montez et al., 2009).

### 4.4 Genetic architecture of spectral *fE/I*

The observed heritability spectra (Section 3.2) indicate a strong genetic contribution to interindividual variance in*fE/I*, which suggests that E/I balance is significantly influenced by genes. This observation aligns with findings on genetically-driven E/I dysregulation and its implications in various brain disorders. For example, in some neurological conditions, such as autism spectrum disorders, epilepsy, STXBP1 syndrome, Rett syndrome, or Fragile X syndrome, genetic alterations affecting synaptic formation, function, or structure are linked to disruptions in excitatory and/or inhibitory signaling, contributing to associated behavioral phenotypes (Fritschy, 2008;Gatto & Broadie, 2010;Houtman et al., 2021;J. Wang et al., 2017). Exploring*fE/I*in relation to disease-specific functional transcriptomic profiles could provide additional insights into potential mechanisms driving E/I imbalances in these disorders, aiding in better patient stratification and the development of personalized treatments.

A similar approach to assessing heritability in narrow frequency bins was previously employed bySmit et al. (2005), where the background EEG power spectrum was found to be strongly influenced by genes, with the highest heritability estimates observed in the alpha frequencies. In*fE/I*, we also observed the strongest genetic effects in the alpha-frequency bins, with significant heritabilities found at both the whole-brain scale and the electrode level. While significant on the whole-brain scale, heritabilities in other frequencies did not reach significance in most individual electrodes (Supplementary Table S1), possibly due to the poor signal-to-noise (SNR) ratio of signals in these bands. A low SNR is known to attenuate DFA exponents (Linkenkaer-Hansen et al., 2007), potentially resulting in an increased number of missing*fE/I*values and reduced statistical power for heritability analyses. In addition, using longer recordings (>5 minutes) may reduce the variance of the correlation estimate and provide a more precise and accurate measure of the true correlation to derive*fE/I*.

The genetic basis of*fE/I*should be investigated in future studies to understand what drives heritability of*fE/I*at different frequencies and how it relates to different network functions. It is plausible that genetic sources reside in the diversity of cellular and functional properties of neurons involved in maintaining or regulating E/I balance within neuronal networks. Inhibitory neurons, for example, exhibit a high degree of heterogeneity in such aspects as morphology, anatomy, electrophysiology, connectivity patterns, circuit affiliation, or response to neuromodulation, which bias their contribution to coordinated network activity and affect cognition and neural dynamics across different brain states (Tremblay et al., 2016). Since brain oscillations in different frequency bands have distinct mechanistic origins (Buzsáki et al., 2013;Lapray et al., 2012), interneuron types may differentially contribute to heritability of frequency bands. In this regard, spectral*fE/I*measurements could help elucidate different functional circuits shaped by molecular and cellular processes within specific contexts.

### 4.5 *fE/I* as indicator of E/I ratio in spectrally- and anatomically-specific brain circuits

In the CROS model,*fE/I*responds similarly to changes in E/I connectivity across the entire frequency spectrum (Fig. 5B). We demonstrate that in the brain,*fE/I*is multidimensional, and alterations in the cortical state, such as during eye-opening, influence E/I ratios within specific frequency components and spatial locations (Fig. 5A).

Previous studies found more sub-critical dynamics during the eyes-open compared to the eyes-closed condition (Bruining et al., 2020;Hahn et al., 2017). In particular, alpha-band oscillations (8–13 Hz) shifted towards a lower*fE/I*during eyes-open rest (Bruining et al., 2020). This modulation in neural dynamics during eye-opening is accompanied by changes in functional connectivity and subjective experience—with eyes-closed dynamics reflecting an inward-focused network with increased multisensory integration and imagination, and eyes-open dynamics reflecting an outward-focused network characterized by increased eye movements and overt attention (Costumero et al., 2020;Xu et al., 2014). Thus, eyes-open-related E/I neuromodulation likely serves as an adaptive mechanism, preparing the brain for cognitive engagement with the external environment (Pfurtscheller, 1992). Our study observed decreased*fE/I*in the alpha range, indicating a decrease in the E/I ratio and a shift to sub-critical network dynamics during eye-opening in neuronal subpopulations underlying alpha activity. This is consistent with the findings of Hahn et al., who found that periods of the eyes-open state were associated with desynchronized cortical states and sub-critical neuronal avalanche dynamics in cat and monkey visual cortices (Hahn et al., 2017), which are the activity hallmarks of the sub-critical regime in the CROS model (Dalla Porta & Copelli, 2019;Poil et al., 2012). While cortical desynchronization is typically associated with enhanced cortical excitability and higher gamma activity during sensory or attentional tasks (Haegens et al., 2011;Harris & Thiele, 2011;Jensen & Mazaheri, 2010;Klimesch, 2012), we did not find changes in*fE/I*in the gamma band in our study. This may be explained by the absence of active cognitive processing during the eyes-open resting state, which would drive the gamma E/I ratio up in an active task (e.g., due to increased network input).

While replicating the widespread decrease in*fE/I*during eyes-open rest in the alpha band (6.5–13 Hz) on a different large cohort of healthy individuals, we identified the peak effect within a narrow range of 8–10 Hz, providing refined spectral specificity and a more detailed characterization of the alpha-band modulation. Additionally, we observed a previously unreported significant reduction in parieto-occipital*fE/I*in the low-beta frequency range (17–22 Hz). Changes in the beta band may similarly be associated with cortical activation (Klimesch, 2012;Pfurtscheller, 2001,^2006^), and the observed effect in 17–22 Hz may suggest a harmonic coupling with alpha, indicating an interaction between these frequency domains (Klimesch, 2012). These shifts in the frequency architecture can reflect cortical preparation for enhanced information processing. Participants, instructed to fixate their eyes on a black cross against a white background during eyes-open rest (see methods inBabayan et al., 2019), displayed a shift to sub-critical beta activity (Supplementary Fig. S5), potentially linked to visual attention and/or motor control associated with maintaining fixation. Overall, these findings highlight the significance of*fE/I*as a valuable indicator of E/I regulation within different neuronal subpopulations.

### 4.6 Practical recommendations

#### 4.6.1 Multiple experimental or behavioral conditions

Alternations between high- and low-amplitude activity could be the natural result of spontaneous fluctuations in critical networks and experimental conditions. Our recommendation would be to control for any behavioral or experimental factors that may induce nonstationarities in the data, for example, where resting-state intervals are interspersed with task intervals. One way would be to annotate data segments. Intervals with the same annotation will be joined into a continuous signal for subsequent DFA and*fE/I*computations. Note that there should be enough data to reliably compute DFA and*fE/I*. Based on preliminary tests, we recommend analyzing at least 2 minutes of activity. In our study, we controlled for these factors. In the twins dataset, all recordings were obtained during eyes-closed rest (Section 2.5.1). In the MPI dataset, recordings were obtained during eyes-open and eyes-closed rest and divided into two conditions for subsequent analyses (Section 2.5.2). All recordings were ≥3 minutes in duration.

#### 4.6.2 Long recordings

For long recordings, such as sleep-EEG or stereo-EEG, we recommend using a windowed approach due to significant state changes, such as transitions between different sleep stages or neuromodulation (figure 5inAvramiea et al., 2022). This approach serves as a useful sanity check before collapsing hour- or day-long recordings into a single value.

#### 4.6.3 Scaling range in DFA

From a statistical physics perspective, it is recommended to infer power-law scaling based on two orders of scaling range (Stumpf & Porter, 2012), which is why early papers on scale-free oscillations used 20-minute long recordings and fitted power laws in DFA, the auto-correlation function and 1/*f*power spectra over time scales up to 200–300 seconds (Linkenkaer-Hansen et al., 2001). For practical purposes, however, many subsequent studies have reported similar findings in shorter scaling ranges (Linkenkaer-Hansen et al., 2007;Monto et al., 2007;Smit et al., 2011). Importantly, fitting DFA to one order of magnitude identifies the critical regime as defined by*k*index and, thus, is not dependent on two orders of magnitude in scaling (Fig. 3G). This is fortunate because recording periods in most neuroscientific experiments are typically in the range of 120–300 seconds. This is relatively short, given the recommendation to include 6–10 independent windows of the longest time scale (Hardstone et al., 2012). For example, for full spectral coverage, lower frequencies are fitted starting at 5 seconds, implying that fitting two orders of magnitude would require fitting to 500 seconds and, thus, more than a 1-hour-long recording. Such long recordings are rare due to practical issues, such as the inability of patients to sit still or stay awake for this long. Our recommendation in the face of limited empirical data is to shorten the fitting range.

### 4.7 Outlook

We believe that the spectrally-optimized*fE/I*methodology will facilitate the interrogation and interpretation of network-scale E/I balance in distinct brain circuitries. Identifying breakpoints in the complex architecture of brain E/I regulation and integrating E/I markers from diverse assessment scales for enhanced clinical decision-making are important aspects of future research. While group-level statistics remain a powerful instrument for analyzing patterns in or differences between conditions, unresolved questions remain as to why some individuals respond or do not respond to treatments. These questions can only be answered by looking at the individual level, allowing us to monitor changes over time, compare reference distributions, and personalize treatment plans. Using*fE/I*as an absolute biomarker of network-level E/I balance, which indicates whether networks are inhibition- or excitation-dominated, may significantly affect the development or choice of drugs given to different subsets of patients. As previously shown inBruining et al. (2020), there is a large variability in*fE/I*in autism spectrum disorder, and further stratification and clinical assessments are needed to understand links to physiological and symptomatic heterogeneities and treatment response (Juarez-Martinez et al., 2023).

## Supplementary Material

Supplementary Material

## Data Availability

The complete MPI Leipzig Mind-Brain-Body dataset is publicly available viahttps://ftp.gwdg.de/pub/misc/MPI-Leipzig_Mind-Brain-Body-LEMON/. The NTR datasets are available on request using the data sharing procedures and forms athttps://ntr-data-request.psy.vu.nl/. The analysis code for this manuscript can be found athttps://doi.org/10.6084/m9.figshare.27102523.v1. The Python implementation of the*fE/I*algorithm is available under a CC-BY-NC-SA license athttps://github.com/arthur-ervin/crosci.
