# Supplementary material for: Functional excitation-inhibition ratio indicates near-critical oscillations across frequencies
Source: Imaging Neurosci (Camb). 2024 Oct 17;2:imag-2-00318. doi: 10.1162/imag_a_00318 (PMC12290852; doi:10.1162/imag_a_00318)
Supplement: Supplementary Material [file imag_a_00318-supp.pdf]

Supplementary Material

Title

Functional excitation-inhibition ratio indicates near-critical oscillations across frequencies

Authors

Marina Diachenko[1], Additya Sharma[1], Dirk Smit[2,3,4], Huibert D. Mansvelder[1], Hilgo Bruining[5], Eco de Geus[6], Arthur-Ervin Avramiea[1,$], Klaus Linkenkaer-Hansen[1,$,*]

*Correspondence: Klaus Linkenkaer-Hansen (klaus.linkenkaer@cncr.vu.nl)

[$] Shared authorship

Supplementary Methods

S1 Detrended Fluctuation Analysis (DFA)

The main steps of the DFA algorithm are as follows:

1. The signal is bandpass filtered in the desired frequency range using a finite-impulse-response (FIR) filter.
2. The amplitude envelope of the filtered signal is extracted using Hilbert transform.
3. Logarithmically spaced window sizes are generated between 0.1 and 1000 seconds, with 20 window sizes for each order of magnitude. The sizes are then converted from seconds to samples using the signal's sampling frequency.
4. The range of times scales of interest is defined and is called the DFA fitting interval. Frequently, the upper bound is set at 30 seconds, as this is the range within which LRTC are typically reported for human brain oscillations (Hardstone et al., 2012). However, it is also constrained by the length of the signal. To have suitable statistics for the largest window size, one may set it to the signal length divided by 10. When choosing the lower bound of the fitting interval, the integration effect of the underlying filters should be considered (Supplementary Methods S3.2).
5. For each window size within the fitting range, the signal profile is calculated and split into 50%-overlapping same-sized windows.
6. The mean standard deviation is calculated to obtain the mean fluctuation per window size.
7. The DFA exponent is estimated as the best-fit line of the mean fluctuation as a function of window sizes in log-log coordinates in the DFA fitting interval.

S2 Functional excitation-inhibition (*fE/I*) algorithm

S2.1 Procedure

The main steps of the *fE/I* algorithm are as follows (Bruining et al., 2020):

1. The signal is bandpass filtered in the desired frequency range using a finite-impulse-response (FIR) filter.
2. The amplitude envelope of the filtered signal is extracted using Hilbert transform.

3. The signal profile is calculated and segmented into 80%-overlapping 5-second windows.
4. The windows are normalized using the mean of the amplitude envelope calculated per window.
5. Subsequently, the normalized windows are detrended.
6. The normalized fluctuation function for each window is computed as the root-mean square fluctuation of the detrended amplitude-normalized signal profile.

Finally, the *fE/I* value is obtained as $1 - r_{W_{amp},W_{nF(t)}}$, where r is Pearson correlation coefficient and W_{amp} and $W_{nF(t)}$ are windowed amplitudes and windowed normalized fluctuations, respectively. The *fE/I* is set to *NaN* (i.e., missing) if the computed DFA exponent does not exceed the DFA threshold of 0.6.

S2.2 Window size for computing *fE/I*

The time window of 5 seconds for *fE/I* calculation was chosen based on several arguments. First, a shorter window (e.g., 5 vs. 10 s) gives higher statistical power for calculating the correlation between amplitude and fluctuation function necessary for deriving *fE/I*, as more windows are available for calculating correlation. It is, however, also essential to choose a window that is not so short that the signal cannot show variation of temporal auto-correlations because of the correlations introduced by the bandpass filters **(Supplementary Fig. S3M)**. Inaccuracies in *nF*(*t*) may, in turn, lead to inaccuracies in the estimation of *fE/I*; since this will limit the range of observed normalized fluctuation values and reduce the range of observed *fE/I* **(Supplementary Fig. S1A),** leading to reduced sensitivity to changes in E/I ratio, when the windows are too short. When the windows are too long, however, the lack of sufficient data points for calculating the correlation becomes an issue for short signals (e.g., 2 minutes). Therefore, the accuracy of *fE/I* is optimal for intermediate values of window size as shown by decreased mean absolute rank change between *fE/I* and *k* for windows in the range of 3–6 seconds **(Supplementary Fig. S1B)**.

S3. Optimizing *fE/I* for application in narrow frequency ranges

For defining the resolution of frequency binning (Supplementary Methods S3.1), 300-second white-signals (*n* = 100) were generated at a sampling frequency of 1000 Hz. The random values were drawn from a normal distribution with the zero mean and the standard deviation of 10 using *numpy.random.normal* function from the *NumPy* package (Harris et al., 2020). For determining the filter correlation bias in DFA and adapting the lower bound of the DFA fitting interval (Supplementary Methods S3.2), we generated 40000 white-noise signals using the same parameters and function.

We used *MNE Python* (Gramfort et al., 2013) implementation of FIR filtering with a Hamming window and default settings of the *filter* function. Specifically, we used “firwin” *fir_design* with “zero” *phase*. To maintain the relative temporal precision across frequencies, the filter order (*filter_length*), by default, was set to 6.6 times the shortest

transition region in seconds. The width of the transition region at the low (*l_trans_bandwidth*) and the high (*h_trans_bandwidth*) cut-off frequency was computed as a multiple of the lower and higher pass-band edges, respectively (refer to *MNE* documentation for the *filter* function). The lower and higher pass-band edges define the frequency range of interest. Here, we optimized the resolution of frequency binning (i.e., *l_freq* and *h_freq*) in the range of 1–150 Hz based on the spectral characteristics of the underlying FIR filters by looking at the trade-off between the spectral resolution and the number of bins (i.e., information redundancy). For this, we used the power spectral density (PSD) of white noise. PSD was computed in the range of 1–150 Hz using Welch's method via the *MNE* function *psd_welch* with the size of a Fast Fourier Transform window of 3000 samples with an overlap of 50%.

S3.1 Defining a fine-grained frequency scale for the spectral *fE/I* algorithm

To determine optimal resolution of bandpass filters, we examined the PSD of FIR filters applied to white noise in the range of 1–150 Hz **(Supplementary Fig. S2)**. To preserve the temporal structure of the signal, which is essential for computing LRTC, the order of the filters is adapted across frequencies such that the temporal resolution of high-frequency activity is higher than low-frequency activity. Consequently, the frequency resolution decreases for higher frequencies, which is reflected in a larger overlap of the power spectra between neighboring frequency bins at higher frequencies **(Supplementary Fig. S2A)**. We can evaluate this overlap for each pair of frequency bins by setting a threshold for the reduction in PSD at 50% and calculating the percentage of overlapping frequency intervals from the spectral resolution of PSD of the two bins at the threshold. For linearly-spaced 1-Hz frequency bins, the percent of frequency-content overlap between the neighboring bins, or spectral leakage, increases with increasing frequency **(Supplementary Fig. S2A–B)**. Defining the frequency bins in the log space reduces information redundancy at higher frequencies but shows increased spectral leakage at lower frequencies especially up to approximately 4 Hz **(Supplementary Fig. S2C)**. To have optimal spectral resolution for both low and high frequencies, we merged the lower frequencies of 1–4 Hz into one bin and used log-spaced binning in the 4–150 Hz range **(Supplementary Fig. S2D)**. This helped avoid superfluous data and delineate the individual frequency components for subsequent analyses.

S3.2 Determining shortest time scales to assess LRTC across frequencies

Filters integrate data over time and, thus, introduce correlations between neighboring samples, which poses a constraint on the shortest time scale at which one can estimate LRTC without filter bias. On the other hand, it is recommended to include time scales as short as technically possible because physiological processes naturally are more auto-correlated on short than long time scales (Linkenkaer-Hansen et al., 2001). Therefore, we scrutinized the temporal correlation bias of the FIR filters by bandpass filtering white

noise signals (*n* = 40000), extracting the amplitude envelope, and computing LRTC using the DFA algorithm **(Supplementary Fig. S3A–H)**.

A set of time scales was defined using logarithmically-spaced values such that 20 bins were available per order of magnitude in the computation range of 0.1–100 seconds. The upper bound of the DFA fitting interval was fixed at 30 seconds. To estimate the time scales where the filters bias DFA exponents, we used the window size bins contained in the range of 0.1–5 seconds. This resulted in 35 combinations for the DFA fitting interval (i.e., [lower bound, 30]). The amplitude envelope of the filtered signal was extracted via Hilbert transform using the *MNE* function *apply_hilbert*.

The expected DFA exponent is 0.5 for white-noise signals on time scales without filter bias **(Supplementary Fig. S3E–I,** *blue***)**. The bending away from this relationship at short time scales indicates that the filter introduces correlation between the samples, leading to an overestimation of the DFA exponent **(Supplementary Fig. S3E–H, S3K,** *red***)**. For higher frequencies, the temporal integration window of the filter gets narrower, and the bending occurs at shorter time scales than for lower frequencies **(Supplementary Fig. S3E–H)**. This indicates that the lower end of the DFA fitting interval can be adapted across frequencies to include shorter time scales for higher frequencies without bias from the filter, which might be useful for capturing auto-correlations in physiological systems oscillating at high frequencies.

To explore this, we first set a threshold as the average of the DFA distribution of white noise across all frequency components (mean = 0.517, standard deviation (SD) = 0.051) **(Supplementary Fig. S3I)**. In this distribution, DFA in each frequency bin was estimated using a fixed DFA fitting interval of 5–30 seconds. Then, we determined how short of a time scale could be used in the fitting of DFA such that the average of DFA exponents in each frequency did not exceed this threshold **(Supplementary Fig. S3M,** *black***)**. This approach only minimally influenced the center of the DFA distribution (mean = 0.523). Importantly, the width of the distribution diminished, hence indicating that the reliability of the DFA estimates improved (SD = 0.040) **(Supplementary Fig. S3J,** *blue* vs. *black***)**. Improved reliability of DFA estimation can be explained by increased statistical power as more data are available to fit a first-degree polynomial when shorter time windows are included into the fitting interval. Shorter time scales have more windows and, thus, greater confidence in estimating the fluctuation function, *F(t)*. In the extreme case, when fitting between 0.1–30 seconds, DFA exponents were inflated since filter-induced correlations considerably influenced the slope of the fluctuation function **(Supplementary Fig. S3M,** *red***)**, which led to a wide distribution with a mean DFA of 0.7 (SD = 0.150) **(Supplementary Fig. S3K,** *red***)** and, hence, more white-noise signals for which *fE/I* was erroneously computed **(Supplementary Fig. S3N,** *red***)**.

An optimal threshold would be neither too stringent, risking the omission of real LRTC, nor too weak, potentially counting spurious LRTC as real. It means that it should include the shortest possible time scales and ignore chance correlation patterns from

white noise. As we saw above, a threshold at shorter time scales may yield a higher mean DFA and produce a narrower distribution **(Supplementary Fig. S3J,** *black***)**, outperforming a threshold at longer time scales which keeps the mean DFA low but has a broader distribution **(Supplementary Fig. S3J,** *blue***)**. To include even shorter time scales and narrow the DFA distribution further, we identified the shortest time scales where the percentage of missing *fE/I* values (because DFA < 0.6) in each frequency component was the highest **(Supplementary Fig. S3N,** *orange***)**. Despite shifting the DFA distribution to a higher mean of 0.538 **(Supplementary Fig. S3L,** *orange***)**, this approach resulted in reduced variability (SD = 0.032). In both approaches (*black* and *orange*), the likelihood of obtaining a value > 0.6 was 0.026 as compared to 0.05 in the conservative fitting approach (*blue*), indicating that less random signals with spurious LRTC will be passed to the *fE/I* algorithm. We opted for the adapted DFA fitting indicated by the *orange* line as it improved the issue of missing values in *fE/I* **(Supplementary Fig. S5)**.

S4 CRitical OScillations (CROS) computational model

In this study, we use the adapted CROS model (Avramiea et al., 2020), implemented using the Brian2 simulator for spiking neural networks (Stimberg et al., 2014; RRID:SCR_002998). As in the original paper (Poil et al., 2012), the model consists of 75% excitatory and 25% inhibitory integrate-and-fire neurons placed on a 50x50 grid **(Fig. 1A)**. Inhibitory neurons are placed first using Mitchell's best candidate algorithm (Mitchell, 1991), then excitatory neurons are added in the remaining spaces. Networks differ in their two connectivity parameters, C_E and C_I, which are the percentage of other neurons within a local range (circle with a radius of 4 neurons centered on the presynaptic neuron) that each excitatory and inhibitory neuron connects to, respectively. Border neurons have fewer connections because these neurons have a lower number of neurons in their local range. Within the local range, connection probability decreases exponentially with distance. More specifically, the probability, *P*, of a connection at a distance *r* is given by:

$$P(r) = \min(\alpha e^{-r}, 1), \qquad (1)$$

where α is optimized separately for excitatory and inhibitory neurons such that the overall connectivity probability within a neuron's local range is equal to C_E or C_I, depending on whether the neuron is excitatory or inhibitory. For example, in the case of an excitatory neuron *i*, with connectivity probability C_E, where the set of neighboring neurons within the local range is *J*, and |*J*| is the number of neighbors, α will have to satisfy the following equation:

$$\sum_{j\in J} P(r_j) = \sum_{j\in J} \min(\alpha e^{-r_j}, 1) = C_E|J|. \qquad (2)$$

As such, we use the Nelder-Mead optimization algorithm to determine the value of α which minimizes the following function:

$$f(\alpha) = \left|\sum_{j\in J} \min(\alpha e^{-r_j}, 1) - C_E|J|\right|. \qquad (3)$$

S4.1 Neuron model

Neurons are modeled using a synaptic model integrating received spikes, and a probabilistic spiking model. Each time step (*dt*) of 1 ms starts with each neuron, *i*, updating the input I_i, with spikes received from the presynaptic neurons J_i, together with an exponential synaptic decay:

$$I_i(t + dt) = \left(I_i(t) + \sum_j^{J_j} W_{ij}\, S_j(t)\right)\left(1 - \frac{dt}{\tau_I}\right). \quad (4)$$

The weights W_{ij} are fixed and depend on the type of the pre- and postsynaptic neuron. τ_I is the decay constant of inputs, and S is a binary vector, with S_j = 1 if the presynaptic neuron *j* fired in the previous time step, and S_j = 0 otherwise.

The activation of a neuron A_i, is then updated with these excitatory and inhibitory inputs, together with an exponential decay, τ_p, and a baseline activation level A_0:

$$A_i(t + dt) = \left(A_i(t) + I_i(t)\right)\left(1 - \frac{dt}{\tau_p}\right) + A_0\frac{dt}{\tau_p}. \quad (5)$$

The spiking probability P_i^S is calculated as a function of the neuron activation *A* at the current timestep, as follows:

$$P_i^S(t) = \{0\ if\ A_i(t) < 0,\ A_i(t)\ if\ 0 \le A_i(t) \le 1,\ and\ 1\ if\ A_i(t) > 1\}. \quad (6)$$

We determine whether the neuron spikes with the probability P_s. If a neuron spikes, the neuron activation *A* is reset to the reset value, A_r. At the next time step, all neurons that it connects to will have their input updated again according to Equation 4.

S4.2 Model parameters

Parametrically, the model is the same as described in the original paper except for the synaptic weights which are optimized for power-law avalanches and LRTC via an evolutionary algorithm (Smit and Eiben, 2011). Neuron model: ($\tau_I = 9\,ms$), Synaptic model: Excitatory neurons ($\tau_p = 6\,ms,\ A_0 = 0.000001,\ A_r = -2$), Inhibitory neurons ($\tau_p = 12\,ms,\ A_0 = 0,\ A_r = -20$). To improve the range and stability of the LRTC from the original model, an evolutionary algorithm is applied to the synaptic weights. The parameters that can vary are the 2 connectivity parameters (taking values between 0 and 100%), and the natural logarithm of the magnitude of the 4 synaptic weights, W_{EE}, W_{IE}, W_{EI}, and W_{II} (taking values between -5 and 1). For each run, a fitness value is calculated based on the avalanche size, k_{size}, and duration distributions, $k_{duration}$, and the LRTCs.

$$fitness = \frac{1}{|1-DFA| + |1-k_{size}| + |1-k_{duration}|}. \quad (7)$$

The optimum weights (W_{ij}, connecting the presynaptic neuron *j* to the postsynaptic neuron *i*) found by the algorithm are (W_{EE} = 0.0085, W_{IE} = 0.0085, W_{EI} = -0.569, W_{II} = -2).

Supplementary Figures

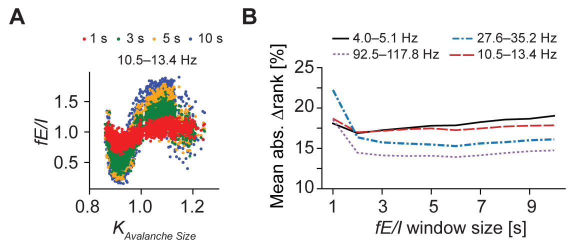

Supplementary Figure S1. *fE/I* accuracy is optimal for intermediate values of window size.
(A) The relationship between *fE/I* (y-axis) and *k* index (x-axis) is shown for four different window sizes in the alpha band. Points represent simulations of 121 CROS networks, generated along a diagonal orthogonal to the critical line with different excitatory connectivity parameters varying from 30% to 42% at 0.1% intervals, and inhibitory connectivity = 80%-excitatory connectivity. Each network was simulated 20 times for 120 seconds (n = 121x20). **(B)** The average absolute rank difference between *fE/I* and *k* ranks (y-axis), Δrank (Section 3.1) is shown for 10 different window sizes (x-axis) in the range of 1–10 seconds, computed in the CROS signals filtered in 4 different frequency bins.

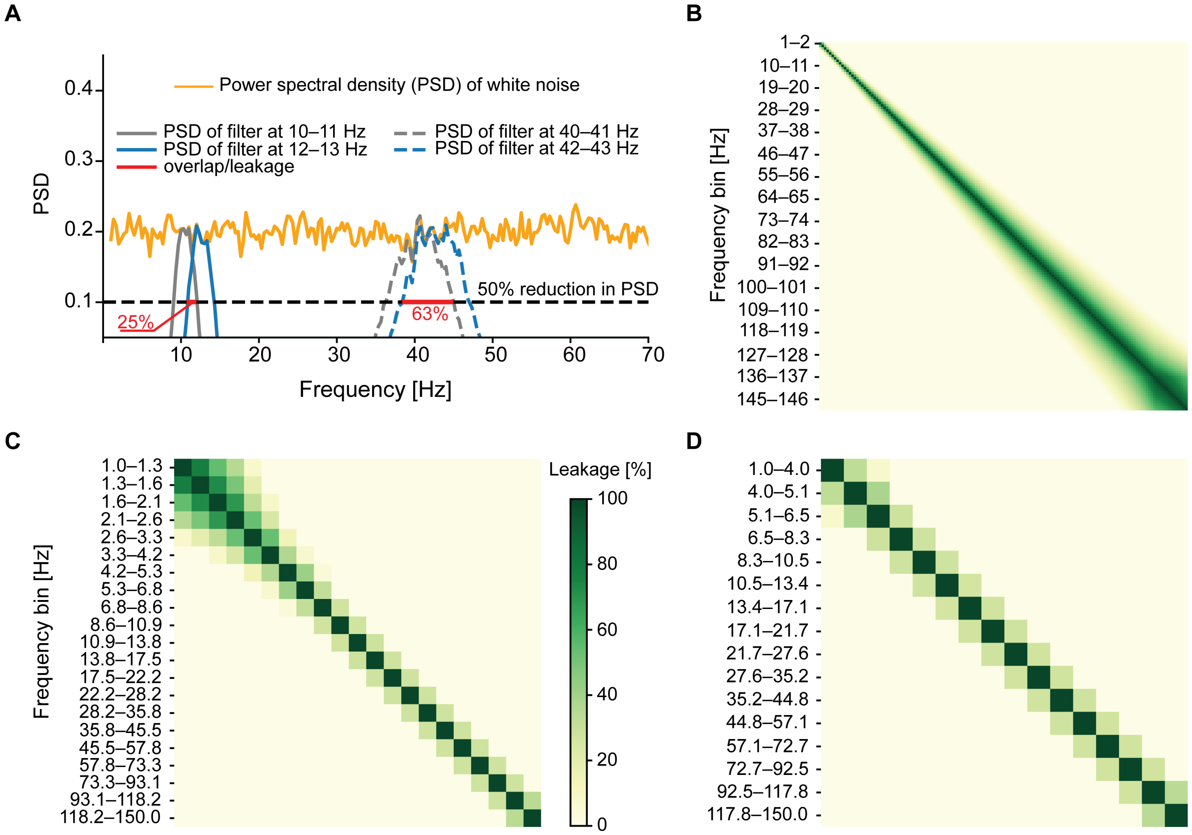

Supplementary Figure S2. Log-spaced frequency binning optimizes the trade-off between the spectral resolution and data redundancy.
(A) The frequency resolution of 1-Hz-range bandpass filters decreases as the frequencies increase (e.g., 10–11 Hz vs. 40–41 Hz). Narrower temporal integration window of the filters at higher frequencies leads to increased overlap (*red*) between the power spectra of the neighboring frequency bins at higher frequencies. PSD of white noise bandpass filtered at 1–150 Hz (*yellow line* shown for 1–70 Hz) and of the same signal filtered at 10–11 Hz, 12–13 Hz, 40–41 Hz, and 42–43 Hz are shown. **(B–D)** The average overlap of power across 100 white-noise signals is shown for three binning strategies. Both axes in each heatmap represent the same set of frequency bins where tick labels are shown only for the y-axis to improve visualization.

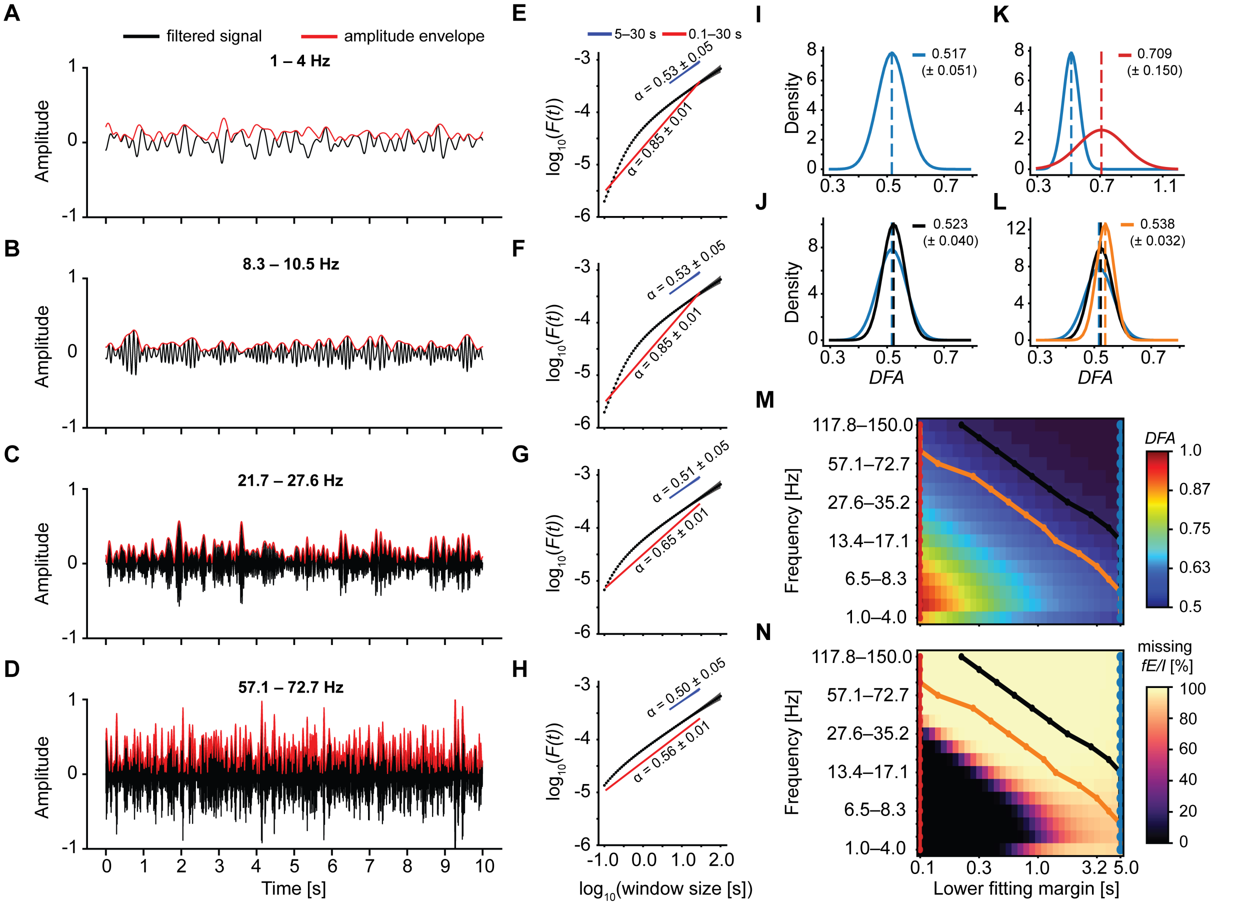

Supplementary Figure S3. Integrating shorter time scales to assess long-range temporal correlations, while mitigating bias of filter-induced correlations, enhances stability in DFA estimates across the frequency spectrum.
(A–D) White noise bandpass filtered in the frequency bins specified in **Supplementary Figure 2D**. **(A)** 1–4 Hz, **(B)** 8.3–10.5 Hz, **(C)** 21.7–27.6 Hz, and **(D)** 57.1–72.7 Hz are shown for one signal. **(E–H)** DFA was performed on the amplitude envelope of the filtered signal, and the mean fluctuation *F*(*t*) as a function of window size was plotted on a log-log axis for each of the frequency bins from A–D, respectively. The average DFA ± standard deviation is depicted next to the corresponding best-fit lines (i.e., *red*, *blue*). **(I–L)** DFA exponent distributions of white noise where the lower DFA bound was set to 5 seconds across all frequencies (*blue*), adapted across frequencies by thresholding the mean DFA in each bin by the mean of the *blue* distribution (*black*), was set to 0.1 seconds (*red*), and adapted by identifying the time scales with the highest percentage of missing *fE/I* values in each frequency bin (*orange*). The means (± standard deviation) are shown. **(M–N)** Color-coded mean DFA and percentage of white-noise signals with missing *fE/I*, respectively, are shown over the spectrum (y-axis) against the lower window of the DFA fitting interval (x-axis). *Blue*, *black*, *red*, and *orange* lines indicate lower windows for the DFA fitting interval described in I–L.

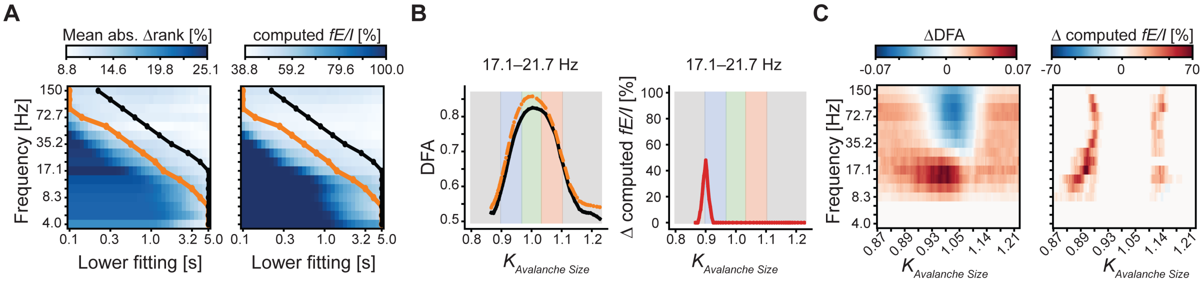

Supplementary Figure S4. Frequency-adapted time scales in the DFA fitting allow more networks to be captured with *fE/I*.

(**A**) *fE/I* accuracy (*left*), measured by the mean absolute rank difference, Δrank, and the number of networks for which *fE/I* can be computed (*right*) are plotted against the lower window of the DFA fitting interval. **(B)** DFA estimated in the range of 17–22 Hz (*left*) using a more conservative fitting approach (*black*) and a more lenient fitting approach is shown for the CROS networks sampled across a diagonal orthogonal to the critical line (Section 2.4, *Validation sample*). The difference in the percentage of computed *fE/I* (17–22 Hz) between the two approaches is shown in *red* (*right*). Networks were arranged on the x-axis based on their corresponding *k* values, and a moving average was calculated using a window size of 50 and an overlap of 80%. Both-side padding with *NaN*s was applied for the Δcomputed *fE/I* values before computing the moving average. Both-side padding for the DFA was applied using the respective first and last values. **(C)** Color-coded DFA (*left*) and percentage of computed *fE/I* (*right*) are shown for the CROS networks across the spectrum (y-axis). A moving average was calculated using a window size of 20 and an overlap of 80%. Padding was performed the same as in B.

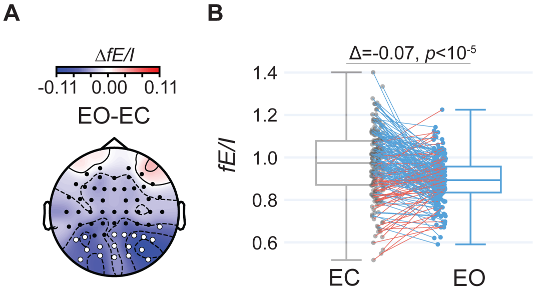

Supplementary Figure S5. Parieto-occipital activity in the beta frequency shifts to sub-critical dynamics during eye-opening.

(A) A significant reduction in *fE/I* in the beta frequency range between 17–22 Hz is observed in the parieto-occipital electrodes in eyes-open (EO) rest compared to eyes-closed (EC) rest. Values represent the mean differences across individual subject *fE/I*s between EO and EC. White circles on the topography indicate significant electrodes based on corrected *p*-values (Bonferroni, across 61 electrodes) for the condition effect after running LMM. **(B)** This reduction indicates a shift to a sub-critical inhibition-dominated regime. A paired *t*-test with significance set at 0.05 was performed on the means of individual subject *fE/I*s across significant electrodes from A. Δ shows the mean difference of EO-EC.

Supplementary Tables

Supplementary Table S1. Heritability of *fE/I* is significant from low to high frequencies. Heritability of *fE/I* for each frequency bin (*column*) and electrode (*row*) as well as of whole-brain average *fE/I* (*last row*) was estimated from an ACE/AE model **(Fig. 2)**. The first row in each cell shows heritability (h^2) in % and the total number of twin pairs with non-missing *fE/I* (*n*). The second row contains the correlation of *fE/I* (*r*) between monozygotic twins and dizygotic twins in the parentheses. The third row displays the mean *fE/I* ± SD across subjects. Significant genetic effects are highlighted in bold. Except for the *fE/I* average across electrodes in the last row of cells, *p*-values across electrodes and frequency bins were not corrected for

multiple comparisons. The significance level was set at 0.05. Significance was determined with likelihood ratio tests. The fourth row in the last row of cells indicates *p*-value for the genetic effects on the *fE/I* average across electrodes, which was Bonferroni-corrected across 9 frequency bins.

Freq. [Hz]		1.0–4.0	4.0–5.1	5.1–6.5	6.5–8.3	8.3–10.5	10.5–13.4	13.4–17.1	17.1–21.7	21.7–27.6
Fp1	h^2	**34%**	33%	**39%**	**36%**	**59%**	**56%**	7%	6%	0%
		**(*n*=60)**	(*n*=50)	**(*n*=64)**	**(*n*=101)**	**(*n*=123)**	**(*n*=151)**	(*n*=101)	(*n*=70)	(*n*=49)
	r	**0.38**	0.47	**0.41**	**0.34**	**0.62**	**0.53**	-0.003	0.12	-0.23
		**(0.13)**	(-0.08)	**(0.18)**	**(0.18)**	**(0.37)**	**(0.36)**	(0.16)	(-0.07)	(0.02)
	fE/I	**0.87 ±**	0.89 ±	**0.86 ±**	**0.98 ±**	**1.12 ±**	**1.02 ±**	0.88 ±	0.92 ±	0.91 ±
		**0.17**	0.16	**0.18**	**0.21**	**0.27**	**0.24**	0.17	0.16	0.15
Fp2	h^2	15%	29%	18%	**41%**	**62%**	**56%**	11%	0%	10%
		(*n*=77)	(*n*=48)	(*n*=73)	**(*n*=101)**	**(*n*=123)**	**(*n*=145)**	(*n*=91)	(*n*=66)	(*n*=49)
	r	0.21	0.59	0.15	**0.39**	**0.67**	**0.56**	0.2	-0.05	0.26
		(0.04)	(-0.04)	(0.16)	**(0.22)**	**(0.31)**	**(0.32)**	(-0.07)	(-0.01)	(-0.02)
	fE/I	0.89 ±	0.91 ±	0.85 ±	**0.98 ±**	**1.11 ±**	**1.02 ±**	0.87 ±	0.91 ±	0.91 ±
		0.17	0.17	0.19	**0.21**	**0.26**	**0.24**	0.18	0.16	0.17
F3	h^2	21%	22%	**38%**	**53%**	**66%**	**61%**	**34%**	9%	0%
		(*n*=59)	(*n*=49)	**(*n*=73)**	**(*n*=102)**	**(*n*=116)**	**(*n*=143)**	**(*n*=123)**	(*n*=95)	(*n*=66)
	r	-0.01	0.35	**0.34**	**0.47**	**0.65**	**0.64**	**0.32**	0.16	-0.08
		(0.43)	(-0.13)	**(0.11)**	**(0.24)**	**(0.41)**	**(0.25)**	**(0.10)**	(-0.04)	(0.09)
	fE/I	0.90 ±	0.91 ±	**0.89 ±**	**0.95 ±**	**1.11 ±**	**0.99 ±**	**0.79 ±**	0.87 ±	0.86 ±
		0.17	0.17	**0.20**	**0.21**	**0.26**	**0.25**	**0.19**	0.17	0.17
F4	h^2	**39%**	6%	**62%**	**43%**	**55%**	**63%**	23%	14%	19%
		**(*n*=60)**	(*n*=45)	**(*n*=63)**	**(*n*=102)**	**(*n*=107)**	**(*n*=134)**	(*n*=121)	(*n*=108)	(*n*=70)
	r	**0.30**	0.05	**0.64**	**0.35**	**0.63**	**0.65**	0.17	0.08	0.26
		**(0.28)**	(0.14)	**(0.14)**	**(0.33)**	**(0.26)**	**(0.20)**	(0.15)	(0.12)	(-0.05)
	fE/I	**0.88 ±**	0.92 ±	**0.88 ±**	**0.93 ±**	**1.07 ±**	**0.97 ±**	0.78 ±	0.86 ±	0.85 ±
		**0.17**	0.18	**0.19**	**0.21**	**0.25**	**0.25**	0.19	0.18	0.16
F7	h^2	0%	10%	**2%**	**44%**	**66%**	**64%**	2%	0%	12%
		(*n*=62)	(*n*=53)	**(*n*=66)**	**(*n*=104)**	**(*n*=115)**	**(*n*=141)**	(*n*=106)	(*n*=64)	(*n*=55)
	r	-0.09	0.11	**0.60**	**0.33**	**0.70**	**0.65**	0.05	-0.19	0.15
		(0.03)	(0.22)	**(0.58)**	**(0.35)**	**(0.30)**	**(0.31)**	(-0.003)	(0.09)	(0.06)
	fE/I	0.86 ±	0.93 ±	**0.89 ±**	**0.98 ±**	**1.11 ±**	**1.01 ±**	0.87 ±	0.92 ±	0.87 ±
		0.17	0.16	**0.17**	**0.20**	**0.26**	**0.24**	0.17	0.16	0.16
F8	h^2	15%	27%	**45%**	**33%**	**52%**	**43%**	15%	25%	0%
		(*n*=74)	(*n*=51)	**(*n*=59)**	**(*n*=90)**	**(*n*=123)**	**(*n*=136)**	(*n*=95)	(*n*=73)	(*n*=56)
	r	0.17	0.30	**0.47**	**0.32**	**0.53**	**0.41**	0.11	0.37	-0.21
		(0.07)	(0.11)	**(0.22)**	**(0.26)**	**(0.39)**	**(0.36)**	(0.18)	(-0.05)	(0.17)
	fE/I	0.88 ±	0.92 ±	**0.88 ±**	**0.95 ±**	**1.08 ±**	**1.01 ±**	0.86 ±	0.88 ±	0.89 ±
		0.16	0.16	**0.17**	**0.20**	**0.25**	**0.23**	0.17	0.16	0.16
C3	h^2	25%	16%	**32%**	**50%**	**55%**	**35%**	8%	18%	19%
		(*n*=53)	(*n*=52)	**(*n*=68)**	**(*n*=92)**	**(*n*=119)**	**(*n*=146)**	(*n*=128)	(*n*=125)	(*n*=115)
	r	0.25	0.22	**0.43**	**0.49**	**0.57**	**0.28**	0.03	0.12	0.23
		(0.14)	(0.03)	**(0.07)**	**(0.26)**	**(0.28)**	**(0.36)**	(0.08)	(0.13)	(0.07)
	fE/I	0.93 ±	0.94 ±	**0.91 ±**	**0.94 ±**	**1.04 ±**	**0.96 ±**	0.87 ±	0.88 ±	0.85 ±
		0.16	0.16	**0.18**	**0.20**	**0.23**	**0.21**	0.18	0.18	0.17
C4	h^2	18%	36%	24%	**57%**	**39%**	**47%**	**29%**	3%	4%
		(*n*=62)	(*n*=57)	(*n*=70)	**(*n*=98)**	**(*n*=127)**	**(*n*=156)**	**(*n*=139)**	(*n*=123)	(*n*=116)
	r	0.19	0.34	0.34	**0.64**	**0.50**	**0.49**	**0.24**	0.15	0.19
		(0.05)	(0.19)	(-0.06)	**(0.20)**	**(0.09)**	**(0.22)**	**(0.24)**	(-0.11)	(-0.22)
	fE/I	0.88 ±	0.93 ±	0.88 ±	**0.92 ±**	**0.99 ±**	**0.94 ±**	**0.84 ±**	0.89 ±	0.87 ±
		0.17	0.16	0.17	**0.19**	**0.23**	**0.22**	**0.17**	0.17	0.16
P3	h^2	30%	**51%**	**47%**	**35%**	**46%**	**41%**	17%	0%	11%
		(*n*=57)	**(*n*=65)**	**(*n*=77)**	**(*n*=109)**	**(*n*=138)**	**(*n*=153)**	(*n*=120)	(*n*=107)	(*n*=100)
	r	0.29	**0.40**	**0.39**	**0.41**	**0.46**	**0.42**	0.27	0.05	0.07
		(0.26)	**(0.24)**	**(0.31)**	**(0.12)**	**(0.40)**	**(0.29)**	(-0.02)	(-0.25)	(0.18)
	fE/I	0.90 ±	**0.90 ±**	**0.87 ±**	**0.94 ±**	**1.05 ±**	**0.98 ±**	0.90 ±	0.92 ±	0.89 ±
		0.17	**0.16**	**0.17**	**0.22**	**0.25**	**0.21**	0.17	0.18	0.15
P4	h^2	3%	**48%**	**37%**	**45%**	**41%**	**34%**	**42%**	19%	0%
		(*n*=82)	**(*n*=68)**	**(*n*=79)**	**(*n*=110)**	**(*n*=131)**	**(*n*=152)**	**(*n*=124)**	(*n*=115)	(*n*=107)
	r	0.13	**0.51**	**0.55**	**0.53**	**0.48**	**0.33**	**0.45**	0.28	-0.06
		(-0.04)	**(0.25)**	**(-0.14)**	**(0.07)**	**(0.15)**	**(0.20)**	**(0.18)**	(-0.04)	(-0.24)
	fE/I	0.86 ±	**0.92 ±**	**0.88 ±**	**0.97 ±**	**1.09 ±**	**0.99 ±**	**0.89 ±**	0.93 ±	0.89 ±
		0.17	**0.16**	**0.18**	**0.23**	**0.25**	**0.21**	**0.18**	0.17	0.17

O1	h^2	10% (*n*=59)	0% (*n*=46)	**28% (*n*=81)**	**41% (*n*=107)**	**56% (*n*=135)**	**50% (*n*=163)**	23% (*n*=112)	**31% (*n*=104)**	**39% (*n*=110)**
	r	0.14 (0.09)	-0.15 (0.07)	**0.26 (0.26)**	**0.44 (0.23)**	**0.62 (0.29)**	**0.50 (0.29)**	0.33 (-0.01)	**0.39 (0.13)**	**0.46 (0.09)**
	fE/I	0.92 ± 0.17	0.94 ± 0.14	**0.93 ± 0.16**	**1.01 ± 0.23**	**1.14 ± 0.28**	**1.07 ± 0.26**	0.95 ± 0.18	**0.96 ± 0.18**	**0.88 ± 0.17**
O2	h^2	**48% (*n*=56)**	26% (*n*=48)	**40% (*n*=63)**	**46% (*n*=99)**	**65% (*n*=123)**	**45% (*n*=157)**	0% (*n*=103)	**40% (*n*=103)**	**26% (*n*=106)**
	r	**0.48 (0.14)**	0.40 (-0.23)	**0.46 (0.02)**	**0.54 (0.28)**	**0.68 (0.32)**	**0.44 (0.31)**	-0.20 (0.02)	**0.41 (0.28)**	**0.38 (-0.01)**
	fE/I	**0.93 ± 0.17**	0.95 ± 0.16	**0.95 ± 0.17**	**1.03 ± 0.23**	**1.17 ± 0.27**	**1.08 ± 0.27**	0.96 ± 0.19	**0.98 ± 0.18**	**0.90 ± 0.18**
T5	h^2	39% (*n*=63)	0% (*n*=49)	**43% (*n*=80)**	**48% (*n*=101)**	**48% (*n*=125)**	**40% (*n*=145)**	14% (*n*=122)	**31% (*n*=86)**	**33% (*n*=87)**
	r	0.23 (0.13)	0.33 (-0.39)	**0.32 (0.43)**	**0.52 (0.29)**	**0.54 (0.23)**	**0.41 (0.32)**	0.26 (-0.06)	**0.46 (-0.08)**	**0.38 (0.08)**
	fE/I	0.91 ± 0.16	0.93 ± 0.16	**0.91 ± 0.18**	**0.97 ± 0.22**	**1.06 ± 0.25**	**0.98 ± 0.22**	0.92 ± 0.17	**0.95 ± 0.18**	**0.91 ± 0.16**
T6	h^2	5% (*n*=76)	26% (*n*=67)	22% (*n*=86)	**44% (*n*=116)**	**55% (*n*=119)**	**44% (*n*=150)**	**28% (*n*=115)**	**28% (*n*=107)**	**33% (*n*=100)**
	r	0.07 (0.01)	0.40 (-0.18)	0.25 (0.09)	**0.44 (0.29)**	**0.59 (0.29)**	**0.45 (0.29)**	**0.24 (0.21)**	**0.25 (0.18)**	**0.35 (0.27)**
	fE/I	0.89 ± 0.17	0.92 ± 0.16	0.92 ± 0.18	**1.02 ± 0.22**	**1.13 ± 0.26**	**1.04 ± 0.22**	**0.96 ± 0.18**	**0.97 ± 0.18**	**0.92 ± 0.17**
Avg eeg	h^2	**39% (*n*=183)**	23% (*n*=180)	**43% (*n*=182)**	**58% (*n*=183)**	**65% (*n*=182)**	**60% (*n*=184)**	**38% (*n*=184)**	21% (*n*=182)	**32% (*n*=172)**
	r	**0.41 (0.13)**	0.31 (0.02)	**0.37 (0.33)**	**0.55 (0.33)**	**0.69 (0.38)**	**0.59 (0.43)**	**0.4 (0.1)**	0.21 (0.07)	**0.39 (0.14)**
	fE/I	**0.90 ± 0.10**	0.94 ± 0.10	**0.91 ± 0.12**	**0.97 ± 0.15**	**1.09 ± 0.21**	**1.00 ± 0.19**	**0.89 ± 0.12**	0.92 ± 0.11	**0.89 ± 0.09**
	p	**3.5e-4**	2.6e-1	**3.1e-5**	**2.3e-9**	**5.6e-14**	**1e-11**	**2.6e-3**	5.4e-1	**3.8e-3**

References

Avramiea, A. E., Hardstone, R., Lueckmann, J. M., Bím, J., Mansvelder, H. D., & Linkenkaer-Hansen, K. (2020). Pre-stimulus phase and amplitude regulation of phase-locked responses are maximized in the critical state. *eLife*, *9*, e53016. https://doi.org/10.7554/eLife.53016

Bruining, H., Hardstone, R., Juarez-Martinez, E. L., Sprengers, J., Avramiea, A. E., Simpraga, S., Houtman, S. J., Poil, S. S., Dallares, E., Palva, S., Oranje, B., Matias Palva, J., Mansvelder, H. D., & Linkenkaer-Hansen, K. (2020). Measurement of excitation-inhibition ratio in autism spectrum disorder using critical brain dynamics. *Scientific reports*, *10*(1), 9195. https://doi.org/10.1038/s41598-020-65500-4

Gramfort, A., Luessi, M., Larson, E., Engemann, D. A., Strohmeier, D., Brodbeck, C., Goj, R., Jas, M., Brooks, T., Parkkonen, L., & Hämäläinen, M. S. (2013). MEG and EEG data analysis with MNE-Python. *Frontiers in Neuroscience, 7*(267), 1–13. https://doi.org/10.1016/j.neuroimage.2013.10.027

Hardstone, R., Poil, S. S., Schiavone, G., Jansen, R., Nikulin, V. V., Mansvelder, H. D., & Linkenkaer-Hansen, K. (2012). Detrended fluctuation analysis: a scale-free view on neuronal oscillations. *Frontiers in physiology*, *3*, 450. https://doi.org/10.3389/fphys.2012.00450

Harris, C. R., Millman, K. J., Van Der Walt, S. J., Gommers, R., Virtanen, P., Cournapeau, D., Wieser, E., Taylor, J., Berg, S., Smith, N. J., Kern, R., Picus, M., Hoyer, S., Van Kerkwijk, M. H., Brett, M., Haldane, A., Del Río, J. F., Wiebe, M., Peterson, P., . . . Oliphant, T. E. (2020). Array programming with NumPy. *Nature*, *585*(7825), 357–362. https://doi.org/10.1038/s41586-020-2649-2

Linkenkaer-Hansen, K., Nikouline, V. V., Palva, J. M., & Ilmoniemi, R. J. (2001). Long-range temporal correlations and scaling behavior in human brain oscillations. *The Journal of neuroscience : the official journal of the Society for Neuroscience*, *21*(4), 1370–1377. https://doi.org/10.1523/JNEUROSCI.21-04-01370.2001

Mitchell, D. P. (1991). Spectrally optimal sampling for distribution ray tracing. *In Proceedings of the 18th annual conference on Computer graphics and interactive techniques (SIGGRAPH '91)*. *Association for Computing Machinery, New York, NY, USA,* 157–164. https://doi.org/10.1145/122718.122736

Poil, S. S., Hardstone, R., Mansvelder, H. D., & Linkenkaer-Hansen, K. (2012). Critical-state dynamics of avalanches and oscillations jointly emerge from balanced

excitation/inhibition in neuronal networks. *The Journal of neuroscience: the official journal of the Society for Neuroscience*, *32*(29), 9817–9823. https://doi.org/10.1523/JNEUROSCI.5990-11.2012

Smit, S., & Eiben, A. E. (2011). Multi-Problem parameter tuning using bones. *Artificial Evolution, 222–223*

Stimberg, M., Goodman, D. F., Benichoux, V., & Brette, R. (2014), Equation-oriented specification of neural models for simulations. *Frontiers in Neuroinformatics, 8:6*. http://doi.org/10.3389/fninf.2014.00006
